# Water Column Correction for Coral Reef Studies by Remote Sensing

**DOI:** 10.3390/s140916881

**Published:** 2014-09-11

**Authors:** Maria Laura Zoffoli, Robert Frouin, Milton Kampel

**Affiliations:** 1 Remote Sensing Division, National Institute for Space Research (INPE), Av. dos Astronautas 1758, São José dos Campos, SP 12227-010, Brazil; E-Mail: milton@dsr.inpe.br; 2 Scripps Institution of Oceanography, UCSD, 9500 Gilman Drive, La Jolla, CA 92093, USA; E-Mail: rfrouin@ucsd.edu

**Keywords:** bottom reflectance, remote sensing, attenuation coefficient, water column constituents, submerse substrate

## Abstract

Human activity and natural climate trends constitute a major threat to coral reefs worldwide. Models predict a significant reduction in reef spatial extension together with a decline in biodiversity in the relatively near future. In this context, monitoring programs to detect changes in reef ecosystems are essential. In recent years, coral reef mapping using remote sensing data has benefited from instruments with better resolution and computational advances in storage and processing capabilities. However, the water column represents an additional complexity when extracting information from submerged substrates by remote sensing that demands a correction of its effect. In this article, the basic concepts of bottom substrate remote sensing and water column interference are presented. A compendium of methodologies developed to reduce water column effects in coral ecosystems studied by remote sensing that include their salient features, advantages and drawbacks is provided. Finally, algorithms to retrieve the bottom reflectance are applied to simulated data and actual remote sensing imagery and their performance is compared. The available methods are not able to completely eliminate the water column effect, but they can minimize its influence. Choosing the best method depends on the marine environment, available input data and desired outcome or scientific application.

## 1. Introduction

Coral reefs are the most biodiverse and productive ecosystems in marine environments [1]. Several studies have shown that these ecosystems appear to be the first to respond to global climate changes, such as increased sea surface temperature (SST) and ultraviolet radiation (UV) and acidification of seawater that results from higher levels of atmospheric CO_2_ concentration. SST increases can lead to a loss of symbiotic relationships between corals and zooxanthells and cause coral bleaching events. In response to ocean acidification, a decrease in the biodiversity of these ecosystems can be expected [2]. Additionally, variation in sedimentation rates caused principally by increases in deforestation can cause negative feedbacks. Because of its sensitivity, coral reefs are considered to act as biological indicators of global climate change [3]. In this context, monitoring programs to detect changes in coral reef biodiversity and coral bleaching are essential.

As in other natural environments, remote sensing approaches to acquiring data in coral reef ecosystems are the most cost-effective and allow for synoptic monitoring of large areas, including places with difficult access [4]. In recent years, studies on coral ecosystems by remote sensing have increased considerably because of a greater availability of orbital sensors with better spatial and spectral resolutions and the development of different methodologies in digital classification processes. Orbital high spatial resolution sensors such as IKONOS and Quickbird (4 and 2.4 m, respectively), high spectral resolution sensors (e.g., *Airborne Visible/Infrared Imaging Spectrometer*—AVIRIS) and other airborne sensors with both high spatial and spectral resolutions (e.g., *Compact Airborne Spectrographic Imager*—CASI, *Portable Hyperspectral Imager For Low Light Spectroscopy*—PHILLS, *Advanced Airborne Hyperspectral Imaging Sensors*—AAHIS) have been used successfully in coral reef studies [5–8], among many others. These technologies have produced improved mapping accuracy compared to other multispectral sensors traditionally used, such as LANDSAT with intermediate spatial resolution.

Bottom reflectance (ρ*_b_*) is the central parameter in the remote sensing of coral reefs and, depends on the physical structure and chemical substrate composition [9]. ρ*_b_* in coral reef studies has been mainly used for the following:

‐ Identification of coral bleaching events, which are frequently used as a proxy for coral reef health [10]. Bleached corals can be differentiated from healthy corals in their reflectance spectrum because the zooxanthells that are lost are associated with pigment depletion and color change [11–13]. Despite its potentiality, it can be complicated to observe by remote sensing and depends on the prompt imagery of the area because dead corals are rapidly colonized by algae, with a spectral behavior similar to zooxanthells;
‐ Mapping of different assemblages of benthonic species by using different techniques, such as methods based on spectra similarities or *Object-Based Image Analysis* (OBIA) [8,14–17], among others. In the latter, knowledge of reflective bands can be introduced, which has resulted in improved mapping accuracy;
‐ Application of spectral mixed indexes to resolve benthic mixtures. This technique has been used in terrestrial environments where the three main fractions considered were vegetation, shadow and soil. In reef environments, it was applied with some success using the fractions algae, coral and sand [7,18,19];
‐ Application of methods such as derivative analysis in quasi-continuous spectra allowing detection of diagnostic features for discriminating between bottom types [3,20,21].

However, in some situations, the actual bottom reflectance spectra are not required. These situations occur when the objective of the work is to solely produce a bottom type map of a coral reef from an individual image using either supervised or unsupervised classification algorithms. In these cases, spectra arising from each mapped class are not valid for descriptive and/or comparative purposes.

Although remote technologies have a great potential in studies of the sea bottom, extracting the reflectance spectrum from the data of orbital optical sensors is complex. Several processes affect the satellite signals, which include four main contributions that should be properly treated. The first corresponds to photons that interact with the atmosphere but do not reach the water surface. This is an inherent problem for any type of terrestrial target studied by remote sensing. Nevertheless, in oceanic environments, atmospheric interference should be carefully considered because Rayleigh scattering caused by gas molecules that constitute the atmosphere is higher in shorter wavelengths where light has a deeper penetration in the water. The second contribution corresponds to photons directly or diffusely reflected by the air-sea interface according to Fresnel laws. The specular reflection of direct sunlight is commonly referred to as the sunglint effect. The amount of energy reflected by the surface depends on the sea state, wind speed and observation geometry (solar and view angles), and in images with very high spatial resolution (lower than 10 m), it causes a texture effect that introduces bottom confusion and distortions in reflectance spectrum [9,22–24]. The third contribution corresponds to photons that penetrate the ocean and interact with water molecules and other constituents of the water column, but do not reach the bottom. The fourth contribution corresponds to photons that have interacted with the bottom and contains information about its reflectance properties. Removing the interference of the atmosphere and surface, the first two contributions, to arrive at the signal backscattered by the water body and bottom requires applying specific procedures (atmospheric correction schemes) to the satellite imagery.

In this study, we leave aside the problem of atmospheric correction and focus on the separation between the signals from the water column and seabed. Among other utilities, this type of correction allows a better discrimination between bottom classes and provides increased accuracy in the classification of digital images [25]. The presentation is organized in three main sections: (i) summary of the main conceptual aspects that refer to water column interferences in shallow submerged bottoms; (ii) compendium of the main methodologies to reduce the water column effect in coral ecosystem studies by remote sensing. Some of the reviewed methods use the TOA signal directly, without specific or prior atmospheric correction; and (iii) application of selected techniques and inter-comparison methods to evaluate their performance to retrieve the bottom reflectance.

## 2. Some Definitions and Concepts in Ocean Color Remote Sensing

### 2.1. Light Penetration in the Water Column

A passive optical sensor in space measures the top-of-atmosphere reflectance (ρ*_TOA_*) (adimensional). Various processes affect the TOA signal, namely scattering and absorption by the atmosphere, Fresnel reflection, backscattering by the water body, and bottom reflection. After crossing the atmosphere to the surface, two distinctions can be made between optically deep water that correspond to water that does not have an influence of the bottom and optically shallow water that is where the remote sensing signal integrates the contribution of the bottom and water column. Water reflectance of the optically deep water (ρ_∞_) and the shallow water (ρ*_w_*), and bottom reflectance (ρ*_b_*) (adimensionals) are defined as:

(1) $$\rho_{w}=\frac{\pi L_{w}}{E_{d0}},\rho_{\infty }=\frac{\pi L_{\infty }}{E_{d0}},\rho_{b}=\frac{\pi L_{b}}{E_{dz}}$$

where *L_w_* is the water-leaving radiance in the presence of the bottom, *L*_∞_ is the water-leaving radiance for an infinitely deep ocean, *L*_b_ is the radiance reflected by the bottom, and *L*_d0_ and *L*_dz_ are the downwelling irradiance at the surface and bottom, respectively. In Equation (1), the bottom and the water body are considered as Lambertian reflectors.

Note that to avoid considering anisotropy of the reflected light field, a commonly used quantity is the remote sensing reflectance (ρ*^RS^*), expressed in sr^−1^. It is defined as:

(2) $$\rho^{RS}=\frac{L_{w}}{E_{d0}}$$

ρ*^RS^* is not *stricto sensu* a reflectance because it has units of sr^−1^. If the water body is Lambertian, then ρ*_w_* and ρ*^RS^* differ by a factor of π.

Reflectance may also be expressed in terms of irradiance. In this case, it is called irradiance reflectance (*R*) (adimensional) and is formally defined as:

(3) $$R=\frac{E_{u}}{E_{d}}$$

where *E_u_* and *E_d_* are upward and downward irradiances, respectively. Depending on the atmospheric correction model applied, results will be in terms of radiance (e.g., water-leaving radiance) or reflectance (e.g., water reflectance or remote sensing reflectance).

In the path between the water surface and marine bottom, electromagnetic radiation interacts with Optically Active Constituents (OAC) by absorption and scattering processes. Both processes occur simultaneously in the water column and can be defined by the beam attenuation coefficient (*c*, m^−1^) as the sum of the absorption (*a*, *m*^−1^) and the scattering (*b*, *m*^−1^) coefficients. The coefficients *a*, *b* and *c* are Inherent Optical Properties (IOP) that depend on the water column characteristics and do not depend on the geometric structure of the light field [26].

Once solar irradiance penetrates the water surface, it decreases exponentially with depth (*z*) according to the Beer-Lambert Law and is a function of wavelength (λ):

(4) $$E_{dz}=E_{d0(z=0^{-})}e^{-K_{d}z}$$

where *E_dz_* and *E_d_*_0(_*_z_*_=0^−^)_ are the downwelling irradiance at depth *z* and just below the water surface, respectively. *K_d_* (m^−1^) is the diffuse attenuation coefficient of the downward irradiance defined in terms of the decrease of the ambient downwelling irradiance (*E_d_*) with depth that comprises photons heading in all downward directions [26]; *K_d_* (λ) varies vertically with depth, but in ocean color remote sensing it is generally used as an average over the first attenuation depth, that is referred to in this work. Unlike *a*, *b* and *c*, *K_d_* is an Apparent Optical Property (AOP) that depends on water column characteristics (scattering and absorption properties) and the geometric structure of light fields.

From *in situ* measurements of the vertical profile of *E_d_*, *K_d_* can be estimated as the slope of the linear regression in a plot of (ln *E_d_*) *versus z* over the depth range of interest. Other approaches that more accurately obtain *K_d_* values may be found in Kirk [27]. *K_d_* can be also estimated from remote sensing data. For example, Lee *et al.* [28] provide an algorithm that performed well even in Case-2 waters (those waters influenced not just by phytoplankton and related particles, but also by other substances that vary independently of phytoplankton, notably inorganic particles in suspension and yellow substances [29–31]. The algorithm is based on estimates of certain IOP, *a* and backscattering (*b_b_*) coefficients obtained from remote sensing using the Quasi-Analytical Algorithm (QAA) [32].

**Figure 1.**
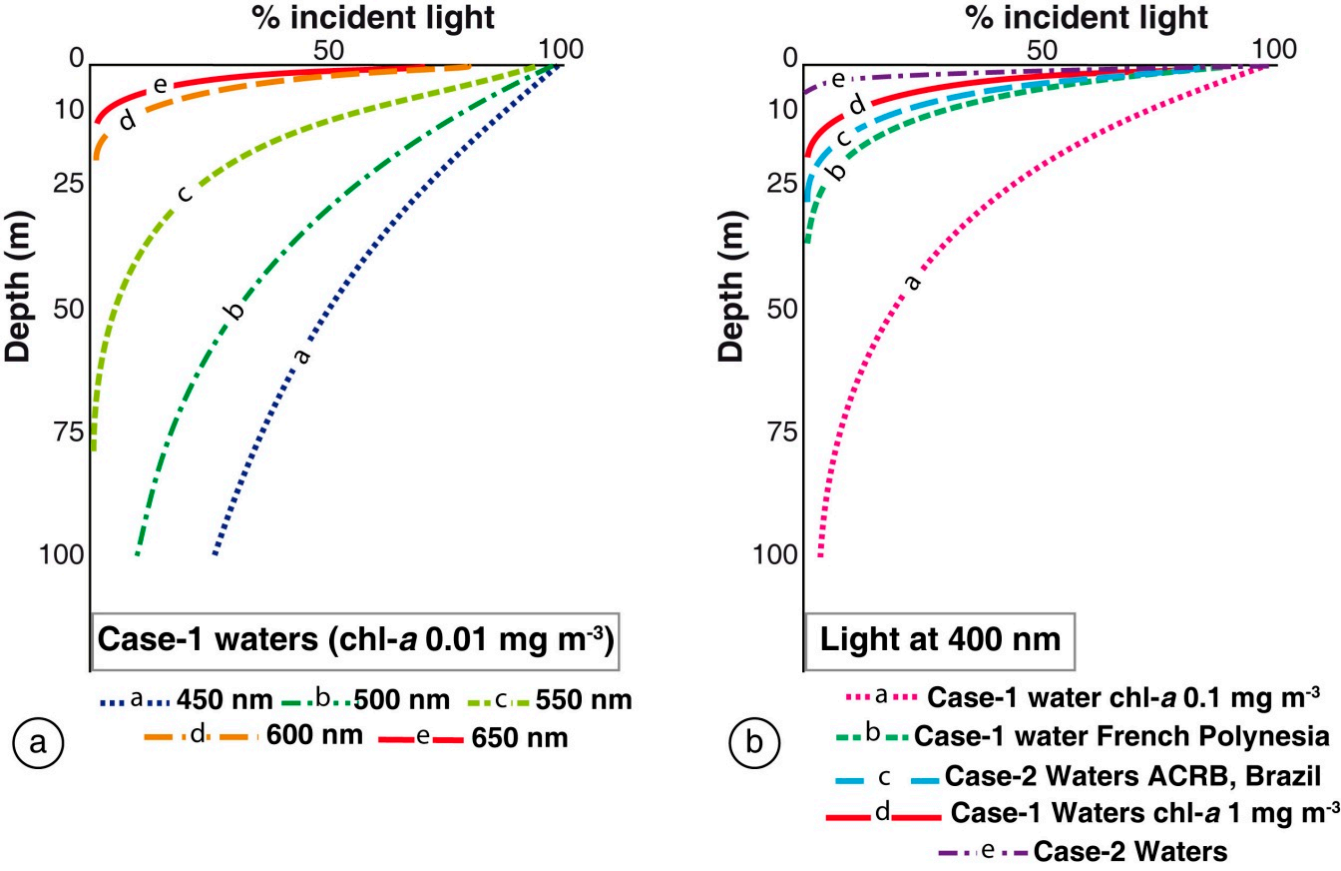
Light decay modeled along water column expressed as percentage of incident light as function of depth (m). (**a**) Curves represent different wavelengths (nm) in an environment considered as Case-1 water, where chl-*a* concentration is 0.01 mg·m^−3^; (**b**) All curves represent light at 400nm but in different kind of environment: Case-1 waters (chl-*a* = 0.1 mg·m^−3^); French Polynesia Case-1 waters (*K_d_* = 0.14 m^−1^); Case-2 waters in Abrolhos Coral Reef Bank (ACRB), Brazil (*K_d_* = 0.18 m^−1^); Case-1 waters (chl-*a* = 1 mg·m^−3^); Case-2 waters (chl-*a*=0.5 mg·m^−3^, *a*_CDOM_ (400) = 0.3 m^−1^, minerals concentration = 0.5 g·m^−3^).

Figure 1a shows the light attenuation in Case-1 waters with very low chl-*a* concentration (0.01 mg·m^−3^) for different wavelengths. In such waters phytoplankton (with their accompanying and covarying retinue of material of biological origin) are the principal agents responsible for variations in optical properties of the water [29–31]. Attenuation increases with λ such that light in the red region has a low penetration, and for this reason in submerged substrate mapping by remote sensing only the visible region is used. *K_d_* increases as the concentration of OAC in the water column increases, making bottom detection more difficult. In low chl-*a* concentration waters (0.10 mg·m^−3^) 3.8% of the irradiance at the water surface penetrates until 100 m depths, but if chl-*a* concentrations rise a 10 fold, attenuation increases disproportionally and this light percentage occurs at only 14 m in depth (Figure 1b). In Case-2 waters with moderate concentration of minerals and CDOM (chl-*a* = 0.5 mg·m^−3^, *a*_CDOM_ (400) = 0.3 m^−1^, minerals concentration = 0.5 g·m^−3^), light penetration decreases to less than 10 m depth in the blue region (400 nm) (Figure 1b).

A maximum depth exists for which a submerged bottom can be detected by optical remote sensing. According to Gordon and McCluney [33], in optically deep waters, the effective penetration depth of imagery (commonly called z_90_) is the layer thickness from which 90% of the total radiance originates; this depth is approximately:

(5) $$z_{90}≅\frac{2.3}{K_{d}}$$

Therefore, if the target of interest is located below z_90_, the water column correction would be severely compromised or not able to correct the water column effect. This would be caused by not enough photons arriving to the bottom and returning to the surface. According to *K_d_* decays, the maximum depth at which a substrate can be detected increases. If the objective is to map a substrate, for example, at 10 m depth, *K_d_* should be equal or lower than 0.10 m^−1^. To map deeper areas, the water should be clearer. The maximum depth at which a substrate can be detected increases as *K_d_* decreases. These depths are further reduced in turbid waters.

### 2.2. Surface and Bottom Reflectance Relation

The reflectance (ρ*_w_*) registered at surface with ρ*_b_* may be related to the water column reflectance following Equation (6) [34]:

(6) $$\rho_{w}=\rho_{\infty }\left(1-e^{-2K_{d}z}\right)+\rho_{b}e^{-2K_{d}z}$$

where ρ_∞_ refers to the water reflectance from optically deep waters *i.e.*, not influenced by the bottom. This reflectance may be approximated by the reflectance of adjacent optically deep waters. Equation (6) indicates that the contribution or the second term to the right-hand side is larger, *i.e.*, the detection of the bottom signal is easier, when *K_d_* and *z* are smaller. Note that the coefficient 2*K_d_* in Equation (6) assumes, for simplification, that the diffuse attenuation for downwelling irradiance is equal to the vertical diffuse attenuation coefficient for upward flux.

Therefore, different substrates (e.g., coral sand, brown algae and green algae) can be easily distinguishable from each other by their spectral behavior when they are at the surface. If the substrates are placed under a clear water column of 1 m thickness, the reflectance will decrease across all spectra, especially at longer wavelengths. This situation would be exacerbated with increments of the bottom depth and, at 20 m, it will be possible to differentiate the substrate type just below 570 nm. If substrates are located in more turbid waters with moderate concentration of Coloured Dissolved Organic Matter (CDOM), their differentiation will be only possible in the green region due to high absorption of CDOM in the blue. Therefore, the remote sensing reflectance should be corrected for the water column effect to minimize the confusion between bottom types caused by differences in depth and OAC.

## 3. Water Column Correction Algorithms

All the water correction algorithms reviewed in the following require data that have been radiometrically corrected/calibrated and masked for land and clouds. Most of them also require previous atmospheric corrections. The algorithms consider the bottom as a Lambertian reflector and the terms reflectance and albedo of the bottom are used interchangeably. They also consider that the signal measured at the surface (being *L*, *R*, ρ*^RS^*) can be separated into two additive components: the water column and the bottom.

Algorithms differ in their ways of estimating partial contributions to the surface signal and we propose grouping them according to their methodological approach. Acronyms, abbreviations and symbols used in this text are made explicit or explained in Appendices 1 and 2. The algorithms are summarized in Table 1, including the approach, characteristics, input data, main equations and output.

### 3.1. Band Combination Algorithms

**Figure 2.**
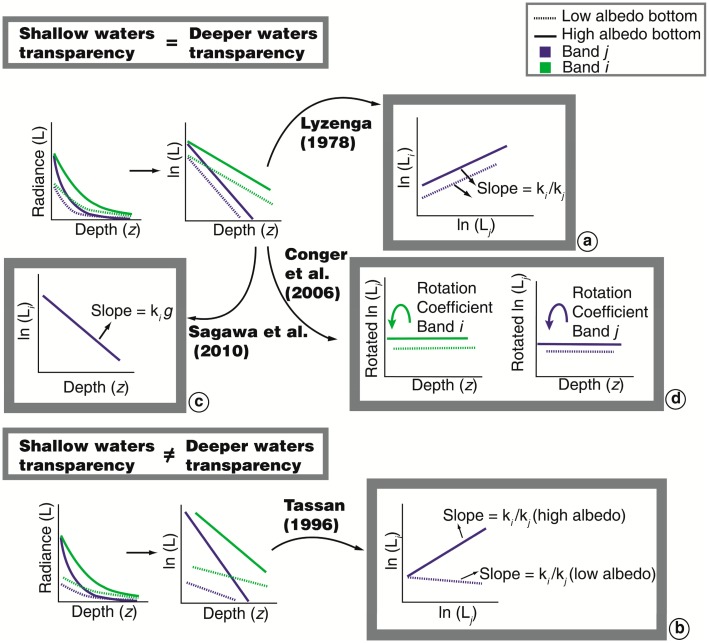
Different strategies proposed to obtain diffuse attenuation coefficient (*K_d_*) from a remote sensing image. These methodologies work with samples of radiance in pixels where depth is known. (**a**) Lyzenga [35]; (**b**) Tassan [37]; (**c**) Sagawa *et al.* [38]; (**d**) Conger *et al.* [39].

Algorithms in this group can be applied to multispectral data and assume that bottom radiance in band *i* (*L_b,i_*) is an exponential function of depth and attenuation coefficient in this band (*K_d_*_,_*_i_*). Given that depth in a pixel is constant for all bands, these algorithms attempt to linearize the relation between radiance in two bands *i* and *j* and water depth [35,36]. The first algorithm was proposed by Lyzenga [35,36] and other derivations have been made and are presented here. Some algorithms use *K_d_*, and although the best estimations of this parameter are obtained from *in situ* data, different approaches to estimate *K_d_* from satellite data have been made (Figure 2).

These algorithms start with *L_TOA_* in shallow areas and the subtraction of *L_TOA_*_,∞_, the deep water radiance. *L_TOA_*_,∞_ accounts for atmospheric influence and water column scattering in deep water. Validity of this procedure for atmospheric correction is limited and sometimes the image to be corrected does not include optically deep waters. An alternative to this procedure could be performing an explicit atmospheric correction on *L_TOA_* and *L_TOA_*_,∞_ and replace (*L_TOA_* – *L_TOA_*_,∞_) by *L_w_* – *L*_∞_.

#### 3.1.1. Lyzenga's Algorithm

Lyzenga's algorithm [35,36] is currently one of the most popular approaches [5,16,17,40–47], among others and the use of this methodology for water column correction has resulted in increased mapping accuracy by digital classification processes [25,48–50]. This is a relatively simple algorithm in which the local depth of the entire scene is not required. The main assumptions are that: (i) differences in radiances between different pixels for the same substrate are due to differences in depth; and (ii) *K_d_* is constant for each band. The first step is to select the pixels samples for the same bottom at different depths and plot (ln(*L_TOA,i_* – *L_TOA,∞,i_*)) *versus* (ln *L_TOA,j_* – *L_TOA,∞,j_*)). The slope of the regression corresponds to a proxy of the attenuation coefficient ratio *K_d,i_*/*K_d,j_* that is a constant value for any substrate. As result, a new image composition of depth-independent composition of corrected radiance in bands *i* and *j* (pseudo-color band) is generated. Figure 2a, adapted from Mumby and Edwards' scheme [51] and Yamano's diagram [52], represents the method proposed by Lyzenga [25]. Because the efficiency of the method relies at least in part on estimating of K*_d,i_*/K*_d,j_* calculated from the scatter plot of corrected radiance *i versus* corrected radiance *j*, chosen samples should correspond to depths for which the remote sensing signal still receives bottom information. This means that for estimating the coefficient ratio in short wavelengths bands (blue and green), bottom depth should be between >0 and 15 m at most. If the ratio needs to be estimated at longer wavelengths (in the red region), bottom depth should be shallower, between >0 and 5 m, because of smaller light penetration in the red.

This algorithm does not retrieve substrate reflectance, instead, the results are a relation between radiances in two spectral bands without a depth effect in (*N* – 1) bands. The algorithm assumes vertical and horizontal homogeneity in optical properties. This method is applicable only in waters with high transparency, and its performance depends on the wavelength. Into the entire visible region, this algorithm produces accurate results until 5 m depth and may be suitable until 15 m depth for bands in the blue and green wavelengths [36]. Lyzenga [36] applied his algorithm to airborne multispectral data and spaceborne Multispectral Scanner (MSS)/LANDSAT data. The validation did not include comparisons with measured reflectance using a radiometer but with percentage of reflectance estimated from color intensity, in pictures registered in 9 homogeneous areas between 3 and 5 m in depths. The remote sensing data corrected with the algorithm yields reflectances with a standard error between 0.018 and 0.036 for the airborne data and MSS/LANDSAT data, respectively.

Mumby *et al.* [25] applied the Lyzenga model in CASI to Thematic Mapper (TM)/LANDSAT, MSS/LANDSAT and Multi-/Satellite Pour l’Observation de la Terre sensors (XS/SPOT) images and then classified the images with and without water column corrections. They recognized that in the CASI image, the water column correction improved the accuracy of the bottom map by 13% in the detailed habitat discrimination, but not in the coarse discrimination. For TM/LANDSAT images, the map accuracy was significantly increased in both, the coarse and fine discriminations. For MSS/LANDSAT and XS/SPOT, the method produced only a single band index using both bands in the visible. The loss of one dimension could not improve the accuracy, even after correction of the deep water effect. In contrast, Zhang *et al.* [17] tested the effect of application of Lyzenga's algorithm in an orbital hyperspectral image of AVIRIS sensor but no accuracy improvement was observed in the map of habitat types. The authors suggested that the low performance of the procedure is because their study area does not present the same substrate in a wide range of depth, which is necessary to obtain accurate values of *K_d,i_*/*K_d,j_*. Therefore, this technique could not be adequate to application in any kind of reef environment. In cases like this, where the same type of substrate only occurs in a narrow range of depths another possibility could be estimation of *K_d,i_*/*K_d,j_* using downward irradiance profiles recorded *in situ* [44]. Hamylton [53] applied Lyzenga's algorithm to a CASI image with 15 spectral bands. She used 28 different band combinations, and even though the optimal combination depended on depth and characteristics of each area, she suggested to:

‐ Maximize the distance between spectral bands used to obtain *Index_ij_*;
‐ Use bands where the light penetrates in all depth range and avoid using bands after 600 nm as a result of the low light penetration in this region;
‐ Use bands with a certain degree of attenuation in the considered depths range to obtain an accurate *K_d,i_*/*K_d,j_*.

##### 3.1.2. Spitzer and Dirks' Algorithm

Spitzer and Dirks [54] developed three algorithms analogous to the one developed by Lyzenga [36] specifically to MSS-TM/LANDSAT and High Resolution Visible/SPOT (HRV/SPOT) sensors. The bands in the visible region of these satellites were renamed as:

(i) Band 1 (Blue region): Band 1/TM (450–520 nm);
(ii) Band 2 (Green region): Band 4/MSS (500–600 nm), Band 2/TM (520–600 nm), and Band 1/HRV (500–590 nm);
(iii) Band 3 (Red region): Band 5/MSS (600–700 nm), Band 3/TM (630–690 nm), and Band 2/HRV (610–680 nm).

The sensitivity of the algorithm to the water column and bottom composition and depth was tested. The *index_b_*_1_ which relates Bands 2 and 3 was limited to shallow waters because bands in the green and red bands in this algorithm have lesser penetration in water. Both *index_b_*_2_ (that consider Bands 1, 2 and 3) and *index_b_*_3_ (which uses Bands 1 and 2) can be used in deeper regions because they consider the blue band. While both *index_b_*_1_ and *index_b_*_2_ can be applied in substrates composed of sandy mud, the *index_b_*_3_ can be used in substrate composed of vegetation and sand. In the three cases, the main limiting factor was the water turbidity [25]. Similar to Lyzenga's algorithm, they do not retrieve substrate reflectance, but the results relate the radiances in two or three spectral bands without a depth effect.

##### 3.1.3. Tassan's Algorithm

Tassan [37] modified Lyzenga's method through numerical simulations for application in environments with important gradients in turbidity between shallow and deep waters. Its sequential application can be described according to the following steps:

‐ Estimate *X′i* = ln[*L_TOA,i_* – *L_TOA_*_,∞,_*_i_*], for two bands *i*, *j* in pixels from two different substrates (e.g., sand and seagrass or high and low albedo, respectively). *L_TOA,∞,i_* corresponds to optically deep TOA radiance, with low turbidity, and *L_TOA,i_* corresponds to shallow TOA radiance, with high turbidity;
‐ Plot *X′_i_ versus X′_j_* for the two substrates and estimate the slope of the linear regressions. Because the slopes of the two lines are different, they did not express a ratio (*K_d,i_*/*K_d,j_*) (Figure 2b);
‐ Perform statistical analysis *X′_ij_* = *X′_i_* – [(*K_d,i_*/*K_d,j_*) (*low albedo*)]*X′_j_* to separate the sand pixels in the shallow waters of seagrass and deep waters pixels;
‐ Perform statistical analysis *X′_ij_* = *X′_i_* – [(*K_d,i_*/*K_d,j_*) (*high albedo*)]*X′_j_* to separate the seagrass pixels.

The result of the algorithm is a relation between two bands; however the real reflectance spectrum is not retrieved. In this work, the algorithm was not applied to real data, so no quantification of its performance was provided.

##### 3.1.4. Sagawa *et al.*'s Algorithm

Sagawa *et al.* [38] developed an index to estimate bottom reflectance based on Lyzenga's method [35,36] that could be applied in environments with low water transparency. For its application, the depth and attenuation coefficient are required. Depth data of various pixels on a homogeneous substrate (sand) allowed estimation of the attenuation. The regression between the radiance and depth of these pixels was calculated (Figure 2c) and the slope of the linear equation corresponded to [*K_d_q*]. In this equation, *q* is a geometric factor that considers the path length in the water column and can be estimated from the angular geometry.

The reliability of this algorithm depends directly on the reliability of Lyzenga's algorithm [36] in which the attenuation coefficient is constant over the entire scene and independent of the substrate type, which may be valid only within small areas. The authors emphasize that the accuracy of the bathymetric map is important for obtaining reliable results. The algorithm application in Case II and III waters, according to Jerlov water types [61] (both correspond to waters with low transparency), increased the accuracy of the classification from 54%–62% to 83%–90%. However, the work of Sagawa *et al.* does not produce an estimation of algorithm efficiency for retrieving bottom reflectance.

##### 3.1.5. Conger *et al.*'s Algorithm

Although Lyzenga's algorithm allows for the removal of the depth effect, after its application, it is difficult to interpret the physics of the pseudo-color images generated by the algorithm. Conger *et al.* [39] proposed linearizing the spectral radiance with depth by application of a principal component analysis (PCA) to estimate the coefficient that allows the signal in each spectral band to be rotated (Figure 2d). The first component explains the higher variability and in this case, represents the signal attenuation that results from increasing depth. The second component provides a coefficient that allows the logarithm of spectral band *i* to be rotated. This procedure generates depth independent data while maintaining the variability caused by small bottom differences.

The algorithm was applied to a multispectral Quickbird image. As result, depth independent pseudo-color bands were obtained that can be calibrated to obtain the bottom albedo, which was performed by Hochberg and Atkinson [62]. Once the application was individually performed for all bands, there was no limitation on their number or width. However, as a result of the low penetration in water of the red wavelengths, this method was not effective for long visible wavelength bands. This algorithm assumes vertical and horizontal homogeneity in the water column optical properties and small albedo variability between samples of the same substrate. Only a visual inspection of the three visible bands of the scene before and after application of the method was performed to evaluate performance.

#### 3.2. Model-Based Algebraic Algorithms

Algorithms in this group require measurements of different water body parameters (e.g., absorption and scattering coefficients) which determine the behavior of light within a water column. Most of the models were not developed to estimate bottom reflectance from surface reflectance measurements, and in general, no validation is provided. Nevertheless, they should be inverted if all other parameters were known.

**Figure 3.**
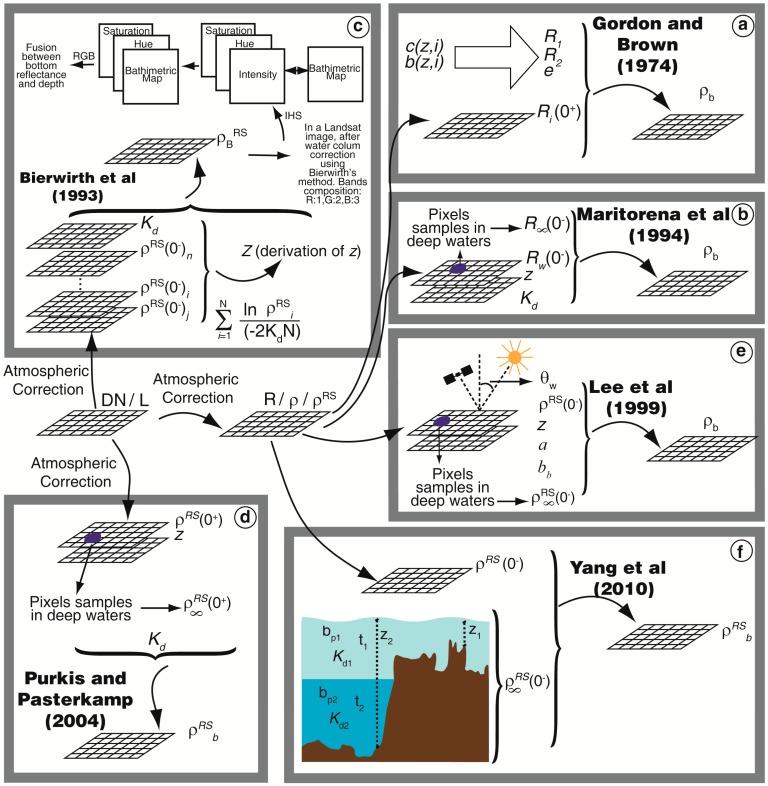
Starting from a remote sensing image above shallow waters, several algorithms can be applied to obtain bottom reflectance. Note that each approach uses distinct inputs. Different boxes represent different algorithms (**a**) Gordon and Brown [55]; (**b**) M94; (**c**) Bierwirth *et al.* [34]; (**d**) Purkis and Pasterkamp [57]; (**e**) L99; (**f**) Yang *et al.* [59].

These algorithms utilize distinctive characteristics of the water column, and parameters used in each method are represented in Figure 3. In the model equations, the parameters are wavelength dependent; for brevity, argument λ was omitted.

For the water column correction of multi-spectral satellite images, *in situ* hyperspectral data used to estimate the parameters required by any model (e.g., *K_d_*, *a*, *b_b_*, *etc.*) must be first integrated over the spectral bands of the sensors [63]. In most cases, the bottom depth is also required. Passive remote sensing in visible bands can be useful when deriving a bathymetric map in shallow clear waters [23,64,65], among others. Also, estimations of the bathymetry using airborne LIght Detection and Ranging (LIDAR) in the blue-green wavelengths can provide highly resolved bathymetric surfaces and offer much greater depth penetration than passive technologies [66].

##### 3.2.1. Gordon and Brown's Algorithm

Gordon and Brown [55] proposed an empirical algorithm that uses three main parameters: *R*_1_, that corresponds to photons that do not strike the bottom; *R*_2_ that represents the contribution of photons to *R_i_* that strike the bottom once for ρ*_b_* = 1; and *s*, the ratio of the number of photons that strike the bottom twice by the number that strike once for ρ*_b_* = 1 (Figure 3a). They depend on the optical depth $\left(\tau_{B}=\int_{0}^{Z_{B}}c(z)dz\right)$, single scattering albedo (*ω*_0_ = *b*/*c*) and scattering phase functions and were provided in Gordon and Brown's work [67]. Phase functions were defined according to the photon path in a water body using Monte Carlo simulations. This algorithm requires some knowledge of the medium characteristics, such as *c* and *b*, however, it was not tested to retrieve the bottom reflectance and its performance was no provided.

##### 3.2.2. Maritorena *et al.*'s Algorithm

Maritorena *et al.* [56] developed a model of the water surface reflectance in shallow waters (*R_w_*) that can be inverted to derivate substrate albedo (ρ*_b_*) from surface measurements. Unlike Gordon and Brown [55], Maritorena *et al.*'s algorithm (hereafter referred to as M94) is a more convenient method based on measurable properties of the water column (Figure 3b). In their algorithm, the irradiance reflectance of shallow waters (*R_w_*) below the surface is equal to the deep water reflectance (*R*_∞_) plus substrate contrast (ρ*_b_* − *R*_∞_) after correction for the depth effect (using the term *e*^[−2^*^K_^d^_z^*^]^). The algorithm's ability to retrieve model *R_w_* was satisfactorily validated with both Monte Carlo simulations and *in situ* measurements. Nevertheless, it was not tested in an inverse manner to obtain the ρ*_b_*.

##### 3.2.3. Bierwirth *et al.*'s Algorithm

In contrast with other methods in this group, Bierwirth *et al.*'s algorithm [34] does not require *z* as input, but only needs ρ*^RS^*(0^−^) and *K_d_*. The method produces results for each pixel; however, it does not retrieve the real bottom reflectance. Derived and real bottom reflectances ( $\rho_{B}^{RS}$ and $\rho_{b}^{RS}$ can be related by a factor (*e*^Δ^*^z^*), where Δ*z* corresponds to an intrinsic methodological depth error. This error is different between pixels but constant between different bands of the same pixel. This means that the properties of the real reflectance can be staggered by the same constant for each band, which varies between pixels. Thus, the spectral hue for each pixel will be preserved, regardless of variations in depth. The authors obtained $\rho_{B}^{RS}$ for the visible bands of a TM/LANDSAT image. For visualization, the $\rho_{B}^{RS}$ values were resampled between 0 and 255. In a composition red-green-blue (RGB) system of $\rho_{B,3}^{RS}:\rho_{B,2}^{RS}:\rho_{B,1}^{RS}$, the observed colors are depth independent. The algorithm was tested successfully and represents a valuable tool for management and analysis of coastal regions and submerged substrates. The authors note that accurate estimates of water column parameters are required and that the model assumes horizontal homogeneity, which may not be valid for certain regions.

If a bathymetric map of the reef is available, this methodology offers an additional utility by producing a fusion to both images. The $\rho_{B,3}^{RS}:\rho_{B,2}^{RS}:\rho_{B,1}^{RS}$ composition can be transformed to an intensity-hue-saturation (IHS) color system. The intensity can be replaced by the bathymetric map, and the layers composition must be transformed again to the RGB system. As result, a fusion image is produced where the bottom reflectance color is preserved and the intensity shows the depth structure of the image (Figure 3c). The orbital image and bathymetric map should be of the same spatial resolution.

##### 3.2.4. Purkis and Pasterkamp's Algorithm

Unlike the other algorithms discussed here, Purkis and Pasterkamp's algorithm [57] considers the refractive effect of the water surface (corrected by the multiplicative factor 1/0.54). Hence, the input of the algorithm is remote sensing reflectance above the surface (0^+^) (Figure 3d). Validation was performed with the radiometric data measured above and below the water over a sand seafloor. The model was able to reproduce *in situ* data with a root mean square (RMS) equal to 0.017. The water column algorithm was applied to a TM/LANDSAT sensor image with different processing levels: (i) homogeneous depth for the entire reef assuming a flat topography; (ii) with a modeled topographic profile; and (iii) depths measured in field. The digital classification showed a higher accuracy for the third case, because of the higher quality of the depth data. Thus, the authors concluded that a bathymetric survey with the spatial resolution compatible with the image spatial resolution is required to produce a map of benthic habitats with sufficient accuracy to be used in quantitative analyses, management or time series studies.

##### 3.2.5. Lee *et al.*'s Algorithm

M94 [56] parameterized attenuation in the water column using a unique parameter *K_d_*. Lee *et al.* [68,58] however, considered that the attenuation coefficients for the upward and downward direction were different and suggested a simple method of estimating them as a function of their IOPs. Lee *et al.*'s algorithm (hereafter referred to as L99), was developed for hyperspectral data, using a water reflectance model based on the quasi-single scattering approximation [69]. Diffuse attenuation coefficients are parameterized as a function of total absorption (a) and backscattering (b*_b_*) (Figure 3e). The inversion scheme retrieves information about water column and bottom properties from spectral data, namely absorption coefficients at 440 nm of phytoplankton (*a_phyto_*(440)), and gelbstoff and detritus (*a_g_*(400)), particle backscattering coefficient at 400 nm (b*_bp_* (400)), bottom reflectance at 550 nm (*B*) and *z*. However, the authors did not compare *in situ* bottom reflectance with those retrieved by the algorithm, but they only used *in situ* values to validate the algorithm for coefficients *a_phyto_* (440), *a_g_* (440) and *z*. Some studies have applied the inversion scheme of L99 to obtain the bottom reflectance, depth and water column properties simultaneously [70–72]. In this scheme, the bottom reflectance (ρ*_b_*) is defined as ρ*_b_* = *B*ρ*_sh_*, where ρ*_sh_* is the albedo shape normalized at 550 nm. Lee *et al.* [71] only considered the shape of the sand albedo; Lee *et al.* [70] also considered the spectral shape of seagrass; whereas Goodman *et al.* [72] used four types of bottom: sand, coral, algae and a flat spectrum. Validations of bottom reflectance included in these studies were limited. Lee *et al.* [70,71] only showed the retrieved bottom reflectances at 550 nm in the form of a histogram or map without comparing them with the ground truth. Goodman *et al.* [72] compared bottom reflectance retrievals with *in situ* reflectance only at 550 nm. They found reflectance estimates are within the range of *in situ* measurements for the majority of the 12 sand substrates used in the validation.

Goodman and Ustin [19] used L99 in both inversion and forward models. First, they inverted the model to obtain the bathymetry and water constituents for all of the pixels from an AVIRIS image. In this step, the bottom reflectance of sand was considered. They found that results were similar regardless of the bottom reflectance spectrum used as input. Once the water constituents and bathymetry were obtained, the authors used this information in a second step as input to the L99 in the forward method together with the reflectance curves of the coral, sand and algae to create end members to apply an unmixing scheme. The unmixing model results were evaluated from depths of 0–3 m and the map accuracy was 80%. The bottom reflectance was also validated at 550 nm for 16 homogeneous sites, and the offset was +10%.

Mishra *et al.* [73] applied L99 [58] to correct a multi-spectral IKONOS image. Because of the limited number of spectral bands of this sensor, the original algorithm was simplified. Application was effective and showed that the differences in radiance between shallow and deeper areas were minimized. The corrected image showed all areas dominated by sand with approximately the same albedo. Only a visual examination of the image after the water column correction was performed. After classification of the corrected image, the map accuracy was 81%.

##### 3.2.6. Mumby *et al.*'s Algorithm

Mumby *et al.* [74] applied a simple model to correct a CASI image of the French Polynesian values. Their model only considered the reflectance at the surface (*R_w_*), *K_d_* and depth for each point of the image, and bottom reflectance was obtained as *ρ_b_* = *R_w_e*^−^*^Kdz^*. The *K_d_* was obtained by the same approach as Lyzenga's, by using the slope of the natural logarithm of reflectance for a uniform substratum (sand) against the depth from ground-truth maps. Derivative analyses were applied to *ρ_b_* spectra, but a validation of the model performance to correct the water column was not provided.

##### 3.2.7. Yang *et al.*'s Algorithm

Unlike the other methods included here, Yang *et al.* [59] developed an algorithm in which the water column is considered multi-layered (Figure 3f). This algorithm can be applied to hyperspectral data and considers water column attenuation and scattering components, both water molecules and other OACs (e.g., phytoplankton and CDOM). The authors applied the algorithm to radiometric data collected *in situ*. For application to orbital or airborne images, a bathymetric map is required. Retrieved values by this algorithm were consistent with *in situ* measured data (fit between retrieved and measured data of *R*^2^ = 0.94). Thus, the algorithm proved to be a robust tool applicable for natural heterogeneous environments that can properly remove the water column influence. However, its application is not simple because thorough knowledge of the environment under study is required to determine the attenuation and scattering coefficients of the OACs and volume scattering functions in each layer of the water column. In addition, the method can be computationally expensive depending on the number of layers. This methodology is suggested for application in environments with strong water column stratification.

#### 3.3. Optimization/Matching Algorithms

**Figure 4.**
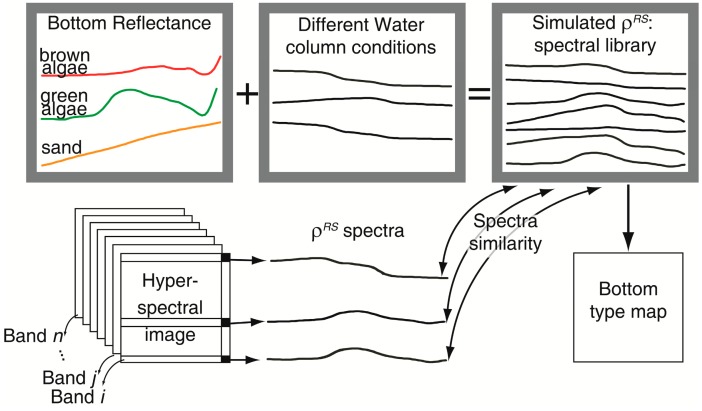
Graphic representation of the approach using Look Up Table matching to generate bottom type map without effect of water column.

Hyperspectral data provides information almost continuous in the visible region that can be able to differentiate between submerged substrates. However, when any of the previous procedures to correct water column effect is applied to images with high spectral resolution, the results are computationally heavy. Another approach that can be used involves simulating the spectra for different water column characteristics and mapping the spectra similarities with the simulated spectral library. The result is a substrate map independent of the water column effect (Figure 4). In other words, the water column effect is “added” to the substrate's underwater spectra and is used for different environmental conditions. Further, classification is performed by assigning to each pixel a substrate type that corresponds to the spectrum in the library that best fits with those in the image. Depending on the proposed algorithm, OAC concentrations and bathymetric map are simultaneously derived. Generation the spectral library requires actual bottom reflectance measured *in situ*. For this reason, all types of substrate reflectances in all possible combinations occurring in the scene must be accurately represented.

Other approach applicable only to hyperspectral data is the inversion scheme in which, using successive runs, measured and simulated reflectance spectra are minimized. Environmental conditions (in this case absorption and backscattering coefficients, depth, bottom reflectance) for which the errors are minimal are considered as the real ones.

For either LUT or inversion methods, the simultaneous retrieval of all the properties (bottom reflectance, depth and water constituents) does mean that the accuracy of each estimated property is highly dependent on the retrieval results for the other properties [75]. The relative effect of each of them depends on the water depth and clarity.

##### 3.3.1. Louchard *et al.*'s Approach

Louchard *et al.* [6] created the spectral library simulated in Hydrolight software using the reflectance of the main bottom types present in the study area, estimations of IOP, *in situ* measurements of upward radiance (*L_u_*) and downward irradiance (*E_d_*), geometric data of the conditions of illumination, image acquisition and range of depth found in the area. The authors also considered Raman scattering. They then applied a minimum distance method to relate the simulated spectra with the spectra of a PHILLS image. This classification methodology generated a thematic map of the substrate without the effects caused by the water column. The authors noted that a good correspondence was found between the classification result and ground truth map, but they did not provide an objective quantification of the accuracy of the substrate type map. The method failed to differentiate dense seagrass from the pavement communities (gorgonians, sponges, hydrocorals, brown and green algae) in areas deeper than 8 m.

##### 3.3.2. Comprehensive Reflectance Inversion based on Spectrum Matching and Table Lookup (CRISTAL)

In contrast to Louchard *et al.*'s approach, Mobley *et al.*'s approach [76] does not require field data and *a priori* assumptions regarding the water depth, IOPs, or bottom reflectance do not have to be made, rather, they are simultaneously extracted from the hyperspectral image. In this approach, pre-computed look-up tables (LUT) are used that include simulated spectral databases generated in HydroLight software for different pure substrates and several combinations of them, in varying depths, OACs in the water column, IOPs, sky conditions and geometry of data acquisition. A total of 41,590 spectra were simulated. The authors only evaluated the method performance by visual interpretation and found that it was successfully applied to a PHILLS image because all variables extracted from the LUT application were consistent with the ground truth. This methodology assumes that the ρ*^RS^* spectrum is accurately calibrated and the spectral library represents all of the environmental variability found in the image. Unlike most of the methodologies, the simulated spectra include inelastic scattering (Raman). If this is not the case, retrieval errors may be large.

##### 3.3.3. Bottom Reflectance Un-Mixing Computation of the Environment Model (BRUCE)

Klonowski *et al.* [77] proposed an adaptation of L99 inversion method [58] to simultaneously retrieve the substrate type and depth from the reflectance collected by the airborne HyMap imaging system (126 bands and 3.5 m of spatial resolution), on the Australian West Coast. In their work, they expressed ρ*_b_* as a linear combination of sediments (*R_sd_*), seagrass (*R_sg_*) and brown algae reflectances (*R_ba_*). Spectral curves of 10 substrate types were used as the input to the model: the six pure substrates of the most frequents in the area (two types of sediments, two types of seagrass and two types of brown algae) and four combinations of them.

For each pixel, the seven unknown parameters *a_phyto_*(440), *a_g_*(440), *b_bp_* (440), *B_sd_*, *B_sg_*, *B_ba_* and *z* are varied to minimize the residual between simulated and measured spectra. As result, three substrate weighting coefficients (*B_sd_*, *B_sg_*, *B_ba_*) are obtained. These coefficients are reflectance scaling factors that represent, after normalization, the proportional coverage by an individual substrate class [78]. They were used to assign the color composition: *B_ba_* to channel red, *B_sg_* to channel green and *B_sd_* to channel blue. The performance validation was performed visually by comparing the mapped substrate with the video records for 25 points of the image, and the authors found a high level of consistency. Although 10 bottom reflectance spectra originating in pure and mixed substrates were considered, in the validation, only 5 classes were considered according to color in the composed image, so different resultant colors might yield the same combination of bottom types and *vice versa*.

Fearns *et al.* [78] applied the BRUCE method in an image collected by the airborne hyperspectral HyMap sensor in the same shallow area. They retrieved proportions of the three classes in each pixel: sand, seagrass and macrophyte species. Map validation was performed to one section of one of the flight lines, and levels of classification success varied according to type of coverage: sand = 52%, seagrass = 48% and brown algae = 88%. The authors suggested that the presence of seagrass at low to medium densities in sand areas could swamp the sand signal and be responsible for low accuracy of the sand class. Higher success to classify brown algae could be related to lower depths where algal habitats were located.

##### 3.3.4. Semi-Analytical Model for Bathymetry, Un-Mixing and Concentration Assessment (SAMBUCA)

Brando *et al.* [79] modified the inversion scheme proposed by L99 [58] to retrieve the bathymetry together with the OAC concentrations (chl-*a*, CDOM and suspended particles) and bottom type. Unlike L99, SAMBUCA accounts for a linear combination of two substrate types. When solving for more than two cover types, SAMBUCA cycles through a given spectral library, retaining those two substrata and the estimated fractional cover which achieve the best spectral fit. The authors were interested in retrieving bathymetric information, and some parameters of the water column were fixed in advance using information collected in field. Based on the types of bottom present in the study area, they only considered brown mud and bright sand. Thus, *g_bm,sand_* is the proportion of the bottom covers. The authors used either least squares minimum (LQM), spectral shape matching function or a hybrid formulation of them to estimate the optimization residuum. Their paper proposed a novel method to improve the bathymetry retrieval by combining the optimization residuum with a substratum detectability index (SDI). Therefore, their focus was to retrieve depths, and they did not provide measurements of performance in retrieving substrate composition.

##### 3.3.5. Adaptive Look-Up Trees (ALUT)

Hedley *et al.* [80] proposed an algorithm that optimizes both the inversion scheme and matching between the simulated and measured spectra to reduce the time required for its application. The Adaptive Look-Up Trees (ALUT) algorithm proposed a more efficient subdivision of the parameter space (any parameter of interest) once the real range of variability is known. Consider changes in the reflectance as a function of depth. It can be observed that in the first depths small changes can lead to greater diminution in measured reflectance. However, at greater depths, small changes lead to lesser impacts in the measured reflectance. Therefore, the ALUT algorithm proposes a more detailed subdivision of the depth in shallow areas than in deeper ones. Additionally, Hedley *et al.* used the matching algorithm Binary Space Partitioning (BSP) tree, which is more efficient than an exhaustive search algorithm.

They used the ALUT approach with the inversion method L99 [58] considering that bottom reflectance spectrum could be one of 78 different curves resulting from the linear mixture of 13 most common substrate types (sand, live and dead coral, algae and seagrass). The method appears to be a promising alternative for rapidly running the water column correction. They obtained high accuracy when retrieving depths from satellite images. However, Hedley *et al.* only compared depths retrieved by the model with depths measured by sonar, and they did not provide an estimation of the efficiency of retrieving bottom reflectance or substrate type compositions. They indicated that their method could have a high level of error when many parameters are derived together.

#### 3.4. Water Column Correction to be Used Only for Multi-Temporal Studies

**Figure 5.**
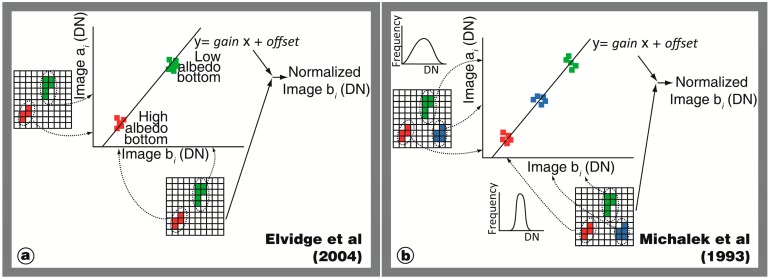
(**a**) Diagram to obtain of gain and offset values to normalized image as function of a previous one, according to Elvidge *et al.* [10] approach; (**b**) Diagram to obtain of gain and offset values to normalize an image as function of a previous one, according to Michalek *et al.* [82] approach.

To detect changes in images from the same sensor in different periods, an inter-calibration between the images is useful. As in all of the temporal studies, co-registration between images must be rigorous because the spatial misregistration can introduce false indications of change. According to Equation (1), if the reflectance of the same invariant target shows differences between two dates, these differences might be caused by the acquisition geometry of a scene, water column and/or atmospheric effects. However, prior atmospheric corrections are not required. One option is the application of a “pseudo-invariant feature” (PIF) technique [10,81] wherein bright and dark pixels (e.g., white beach sand and seagrass substrate, respectively) called PIF pixels, are extracted from all images. Any image can be selected as the reference (Image a) and the other images (Image b) are normalized to be compatible radiometrically with Image a. For this conversion, the digital number (DN) of PIF pixels of Image b are plotted *versus* those of Image a (Figure 5a) in each band. Fitting a linear equation to this plot defines a gain and offset to normalize Image b. This method works under the assumption that PIF pixels are constant over space and time. This type of methods has the advantage of being inexpensive, requiring a small amount of processing time and depending little on data availability

A similar normalization was used by Michalek *et al.* [82] where the image used as the reference (Image a) showed the highest and widest data range in its histogram. Image b was modified to be compatible radiometrically with Image a (Figure 5b). The authors examined pixel samples that appeared similar in natural color in both images, such as bare soil, mangrove forest and deep water.

#### 3.5. Bertels et al.'s Approach

After unsuccessful application of L99, Bertels *et al.* [60] selected another approach to minimize the class confusion caused by the depth effect in CASI images of a coral reef area. They classified an image previously divided by the five geomorphologic zones found in the scene. For the geomorphologic zone mapping, they applied a minimum noise fraction (MNF) analysis to remove redundant spectral information and used the first five bands. Then, independent classifications according to its geomorphology were applied under the assumption that each geomorphologic zone has different depth and associated benthic communities. A post-processing was finally performed to merge the classes between the different zones. The method was only validated their method in the fore reef and obtained an accuracy of 73%. No bottom reflectance spectrum is retrieved by this method, which can only be used in reefs with a determined configuration where the substrate types and geomorphologic zones are strongly related.

### 4. Inter-Comparison of Methods

Bejarano *et al.* [83] applied two methods to minimize the water column effect. They selected Lyzenga's and Mumby's algorithms for the blue and green bands of an IKONOS image to correct the water column effect. After applying the algorithms, they created a habitat map through an unsupervised classification. The overall accuracy obtained from Lyzenga's method was 56%, whereas the Tau coefficient (which characterizes the agreement obtained after removal of the random agreement expected by chance) was 0.43. However, using the individual bands corrected by the Mumby's algorithm, the overall accuracy was 70% and the Tau coefficient was 0.62.

Dekker *et al.* [75] provided the first exhaustive study to compare optimization/matching algorithms to retrieve the bottom reflectance simultaneously with bathymetry and water constituents with high spectral and spatial resolution of airborne images. Their study was conducted at the Rainbow Channel, Moreton Bay (MB) (Brisbane, Australia) using images obtained from the airborne hyperspectral CASI sensor, and at Lee Stocking Island (LSI), (Bahamas) using a PHILLS image. LSI has very clear water conditions, whereas the water column at MB is characterized by spatial heterogeneities. The methods tested were SAMBUCA (using a combination of two substrates), BRUCE (using a combination of three of the most common substrates: sediment, vegetation and coral), CRISTAL (using different substrate combinations, with 39 bottom spectral curves for LSI and 71 for MB) and ALUT (using all possible combinations of the same substrates from SAMBUCA). Validations were performed exclusively between 0 and 3 m in depth. Two approaches were used to evaluate the models' performance in retrieving the bottom type. The first approach compared the *in situ* spectrophotometric measures of ρ*_b_* for the most common bottom types with some points in the images that contained these substrates. The second approach, applied only to the MB area, used reflectances retrieved for each method to produce 2 maps: (i) four classes of seagrass percent cover and (ii) benthic cover and substrate types. Both types of maps were compared with field data. For the first type of validation, only a qualitative adjustment was performed. At LSI, all of the methods retrieved the shape and magnitude of the seagrass spectra, but only ALUT fit the coral shape, and all methods underestimated the sand reflectance. At MB, the results were slightly worse for each substrate type. For both study areas, no method except ALUT reproduced the reflectance peaks and depressions associated with the seagrass spectra. For the second type of validation, the overall accuracies of the seagrass coverage maps were the reference map at 89%, ALUT at 79%, BRUCE at 84%, CRISTAL at 83% and SAMBUCA at 59%. The overall accuracies of the benthic substrate maps were the reference map at 89%, ALUT at 78%, BRUCE at 79%, CRISTAL at 65% and SAMBUCA at 52%. Broadly speaking, the best results were obtained for the most complex and locally parameterized methods, such that there was a more accurate retrieval of reflectance spectra shape and higher map accuracy. ALUT, CRISTAL and BRUCE allowed more detailed retrievals, whereas SAMBUCA was limited to only 3 possible components. The authors noted that effective atmospheric and air-water interface corrections are required to retrieve reliable ρ*_b_*. In terms of practicality, ALUT and BRUCE were the fastest methods considering both the preprocessing and processing times, followed by CRISTAL and the SAMBUCA.

In this section of the article, we present results of a limited inter-comparative analysis of three methods, M94 [56], L99 [58] and CRISTAL [76], with the two first belonging to the second group and the last one to the third group. We did not test any method included in the first group because they produce an index that involves two spectral bands instead of reflectance, *i.e.*, they are not directly comparable in terms of performance with the methods of other groups. Methods proposed by M94 and L99 are similar, yet they differ in that M94 uses an AOP (*K_d_*) to characterize the water column while L99 uses two IOPs (*a* and *b_b_*). These two methods were created to simulate or *ρ^RS^* (0^−^) in shallow waters. They were not tested previously to obtain *ρ_b_* from *ρ^RS^* (0^−^) or *R*(0^−^). The last method (CRISTAL) is a new approach that has the potential to correct the water column. We chose this method as a representative of the third group, and previous studies have already compared different methods of this group [75]. The inter-comparison was accomplished using simulated spectra and multispectral data obtained from the WorldView-2 (WV02) sensor.

#### 4.1. Application and Comparison of Selected Methods: Simulated Spectra

Simulations were performed using the WASI v.4 software [84]. Three types of bottoms were used in the simulations: coral sand, brown algae and green algae [56]. Four depths were considered: 3, 5, 10 and 15 m. Four types of water were defined as representative of the variation of conditions in the water column in coral reefs (Table 2). To define the water constituents, the literature was reviewed to determine the range of variation of chl-*a*, CDOM absorption (*a_CDOM_*) and sediments concentration in coral reefs environments. These parameters vary between chl-*a*: 0.01–9.21 mg·m^−3^; *a*_y_(440): 0.0017–0.24 m^−1^; and sediments: 0.8–2.2 mg·L^−1^. The viewing angle was set to nadir (0°). The solar zenith angle was set to nadir, but the code does not use a relative azimuth angle. In total, 48 situations were considered from the combination of 3 bottoms, 4 depths and 4 waters (3·4·4). In WASI, we simulated the *R*(0^−^), *R*_∞_(0^−^), *K_d_*, ρ*^RS^* (0^+^), $\rho_{\infty }^{RS}(0^{+})$ and *a* coefficients. The selected algorithms were applied to the 48 simulation spectra of *R*(0^−^).

M94 was applied to the 48 simulated spectra of *R*(0^−^) using Equation (7). We excluded in the analysis the cases where ρ*_b_* contribute to *R*(0^−^) with less than 0.5% (Equation (8)) or when ρ*_b retrieved_* behaved exponentially. In all cases, a bottom contribution to the *R*(0^−^) modeled signal could be found even when the bottom was deeper than z_90_:

(7) $$\rho_{b\text{retrieved}}=\frac{R\left(0^{-}\right)-R_{\infty }(0^{-})}{e^{-2K_{d}z}}+R_{\infty }(0^{-})$$

(8) $$\%\text{of bottom contribution}=\frac{(\rho_{b\text{retrieved}}-R_{\infty }(0^{-}))e^{-2K_{d}z}}{R\left(0^{-}\right)}\cdot 100$$

Uncertainties in retrieving the bottom reflectance for each wavelength were estimated as:

(9) $$\text{Uncertainty}=\frac{\rho_{b\text{retrieved}}-\rho_{b}}{\rho_{b}}\cdot 100$$

L99 was applied using Equation (10). ρ*^RS^* (0^+^), $\rho_{\infty }^{RS}(0^{+})$and *a* coefficients spectra were used. ρ*^RS^* (0^+^) and $\rho_{\infty }^{RS}(0^{+})$ were converted to below water using Equation (11).

(10) $$\rho_{b\text{retrieved}}=\frac{\pi \left[\rho^{\text{RS}}(0^{-})-\rho_{\infty }^{\text{RS}}\left(0^{-}\right)\left(1-e^{\left[-\left(1+D_{u}^{C}\right)kz\right]}\right)\right]}{e^{\left[-\left(1+D_{u}^{B}\right)kz\right]}}$$

(11) $$\rho^{RS}\left(0^{-}\right)=\frac{\rho^{RS}(0^{+})}{0.5+1.5\rho^{RS}(0^{+})}$$

The backscattering coefficient is not an output of WASI software. Hence, the *b_b_* coefficients were obtained according to Gege [76]:

(12) $$b_{b}=0.00144{\text{m}}^{-1}{\left(\frac{\lambda }{500}\right)}^{-4.32}+C\text{hl}\_a\left(0.0006{\text{m}}^{2}{\text{g}}^{-1}\cdot C\text{hl}\_a^{-0.37}\right)+C_{\text{Mie}}0.0042{\text{m}}^{2}{\text{g}}^{-1}{\left(\frac{\lambda }{500}\right)}^{n}$$

where *C_Mie_* is the concentration of suspended particles of Type II and *n* is the exponent of backscattering by small particles. We also excluded values of ρ*_b retrieved_* for which bottom contributions were lesser than 0.5% (Equation (13)) or where the results of the model showed an exponential behavior:

(13) $$\%\text{of bottom contribution}=\frac{{\pi \rho }_{\text{bretrieved}}\cdot e^{-\left(1+D_{u}^{B}\right)kz}}{\rho^{\text{RS}}}\cdot 100$$

Uncertainties in retrieving the bottom reflectance for each wavelength were estimated using Equation (9).

Because estimating the parameters used as input in any model involve errors, there are other output uncertainties related to this source. A sensitivity analysis can measure the impact of uncertainties for a parameter on a model result. This type of analysis shows the parameters for which more attention should be paid, because errors in their estimation can cause a significant and non-proportional response in the results. In the analysis, ρ*_b retrieved_* estimated using M94 and L99 from *R*(0^−^) or ρ*^RS^*(0^−^) modeled in WASI constituted the baseline retrieval. Each parameter (*z*, *K_d_*, *R*_∞_, $\rho_{\infty }^{\text{RS}}\left(0^{-}\right)$, *a* and *b_b_*) was then varied between −95% and 100% to evaluate the impact on the ρ*_b retrieved_*. Sensitivity was expressed as follows:

(14) $$\text{Sensitivity}\left(\%\right)=\frac{\left|\rho_{b1}-\rho_{b2}\right|}{\rho_{b1}}100$$

where ρ*_b_*_1_ corresponds to the value obtained from the baseline retrieval, and ρ*_b_*_2_ to the corresponding value after modifying the parameters. This analysis was performed for some extreme cases: Water-a and water-d; sand and brown algae; 3 and 10 m depth. Results were evaluated at 450, 550 and 650 nm.

The CRISTAL method was applied to noisy reflectance spectra. 5% of uncorrelated noise was added to the 48 ρ*^RS^*(0^+^) spectra. Other ρ*^RS^*(0^+^) spectra were also used, and they correspond to various combinations of the three bottoms (coral sand, green algae and brown algae) at 16 depths (1–16 m) and water column constituents (chl-*a*: 0.01, 0.02, 0.1, 0.2, 0.3, 0.9, 1, 1.1, 2.5, 2.8, 3, 3.1, 3.2, 8, 8.5, and 9 μg·L^−1^; *a*_CDOM_(440): 0.0017, 0.00269, 0.0074, 0.1, 0.15, 0.2, and 0.25 m^−1^; concentration of suspended particles Type II: 0, 0.5, 0.8, 1, 2, 2.2, and 2.5 mg·L^−1^). In total, 26,352 spectra were generated and included in the spectral library. The classification technique *Spectral Angle Mapper* (SAM) [85] based on the geometric proximity of two spectra was applied.

For the baseline retrievals using M94 and L99, a gradual loss in bottom contribution was observed for increases in the OAC concentrations and depth. This occurred because when the depth increased, the optical path was augmented, and there were more chances for a photon reflected by the bottom to be absorbed before arriving to the surface. All types of water showed bottom contribution when they were located at 3 m depth. When the bottoms were located at 5 m in water-d, they only had a small contribution to surface reflectance (up to 2% depending on the type of substrate) after 470–500 nm. In contrast, the bottom at 10 m only had contributions from the clearer waters (types a and b) and, in water-c, they were lower than 1% in certain portions of the spectrum. At 15 m, the bottom signal arrived to the surface in the entire visible spectrum only in waters-a and -b up to 600 nm. Additionally, the bottom contribution depended on the physicochemical and biologic characteristics of the substrate. If the bottom is more reflective, it is expected that more photons will come from the bottom and an additional quantity of them will have a chance of crossing the water column and arriving to the surface. For example, for a twice as reflective bottom, its contribution to the surface signal will be higher than for a less reflective bottom, although it will not be proportional.

At some depths, retrievals using M94 displayed anomalously high values when the term *e*^−2^*^k_^d^_z^* tended to zero (e.g., high *K_d_*). Therefore, in waters-a to -c an exponential behavior was observed only in the red region. However, for water-d, this situation was observed also in the blue in response to increasing *K_d_* by CDOM absorption. Retrievals using the L99 also exhibited an exponential behavior in some cases. Generally, this behavior was found when the term $e^{\left[-\left(1+D_{u}^{B}\right)cz\right]}$ was lower than 0.0002.

**Figure 6.**
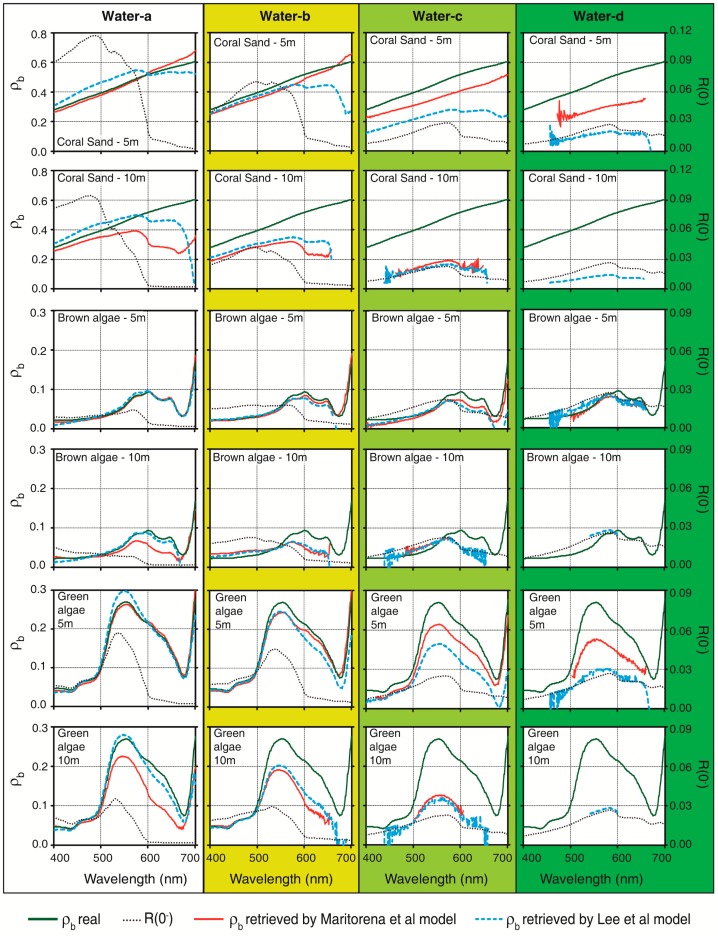
Bottom reflectance below water *versus* wavelength (nm) retrieved using L99 (in **blue**) and M94 (in **red**), compared with real bottom reflectance. In lines, there are results for the same type of substrate and depth. In columns, there are results for the same kind of water.

Note that in the baseline retrievals, *K_d_*, *z*, *b_b_*, *a*, $\rho_{\infty }^{\text{RS}}(0^{-})$, ρ*_b_* and *R*_∞_(0^−^) are known and fixed in advance. Therefore, if the M94 and L99 were used instead of the WASI software to generate *R*(0^−^) or ρ*^RS^*(0^−^), ρ*_b retrieved_* would have been exactly the same as ρ*_b_* in the shallow and clearer waters. Differences between the real and retrieved bottom reflectance by both models are essentially due to differences between the model used by WASI and the tested models. In many cases, M94 could retrieve the shape of the ρ*_b_* spectra from the surface spectra simulated with WASI software (Figure 6). As expected, the algorithm had a better performance in shallower depths and clearer waters. For example, at 5 m depth, the model produced good results up to 700 nm with average of uncertainties of 7% (results are a bit degraded above 600 nm) but performance became degraded above 600 nm when the depth was 10 m (average of uncertainties 44%). The performance was further degraded in water-c and -d, being possible to retrieve ρ*_b_* only in some section of the spectrum depending on the depth, with mean uncertainty of 35%. However, L99 showed a slightly lower performance than M94 (mean uncertainty at 10% for water-a, 5 m; 28% for water-a, 10 m, between 600 and 699 nm) and tended to underestimate the bottom reflectance, especially after 600 nm (Figure 6). This algorithm was able to retrieve the shape of the algae spectra in most cases between 400 and 600 nm in water-a and -b at 5 m and below 600 nm in water-c at 10 m. If *R*(0^−^) was used as the starting point for both models and if *R*(0^−^) was divided by π to obtain ρ*^RS^*(0^−^) according to L99 [58], closer values were obtained between the results of both models.

Reflectances, absorption and backscattering coefficients simulated by WASI software were slightly noisy. In cases where the water reflectance was lowest (red and blue regions in the most turbid waters) this noise was magnified and explains some noisy behavior in the retrievals for certain waters and regions of the spectrum (Figure 6).

The M94 [56] yields errors up to 66% in the clearest waters (water-a) in the range 400–499 nm, 62% in the range 500–599 nm, and 91% in the range 600–700 nm, depending on the bottom depth. The figures become 66%, 21%, and 36%, respectively, when using the L99 [58], which indicates reduced uncertainties as a result of differences in radiative transfer modeling. When waters are more turbid (water-b, -c and -d), the errors generally increase. In the most extreme case (water-d), the errors could be as high as 300% for both models, depending on the portion of the spectrum and bottom depth. In general, there was no pattern associated with uncertainties because they were simultaneously related to the optical path length (2K_d_ · z) and bottom reflectance in a non-linear way.

For M94, however, uncertainties at the shallowest depth appeared to be more sensitive to the variability of the optical path length for the three types of bottoms. For optical path length increases, uncertainties were more related to the reflectance at the water surface. Using L99, uncertainties were not sensitive to a unique input, optical path length, ρ*_b_*, ρ*^RS^*(0^−^) or $\rho_{\infty }^{RS}(0^{-})$, which made it more difficult to predict the model performance in a particular environment. The pattern of uncertainties was also dependent on the bottom reflectance. For example, for the brown algae bottom, uncertainties were not related to a sole factor. In contrast, uncertainties were explained mainly by the optical path length when the bottom was sand.

**Figure 7.**
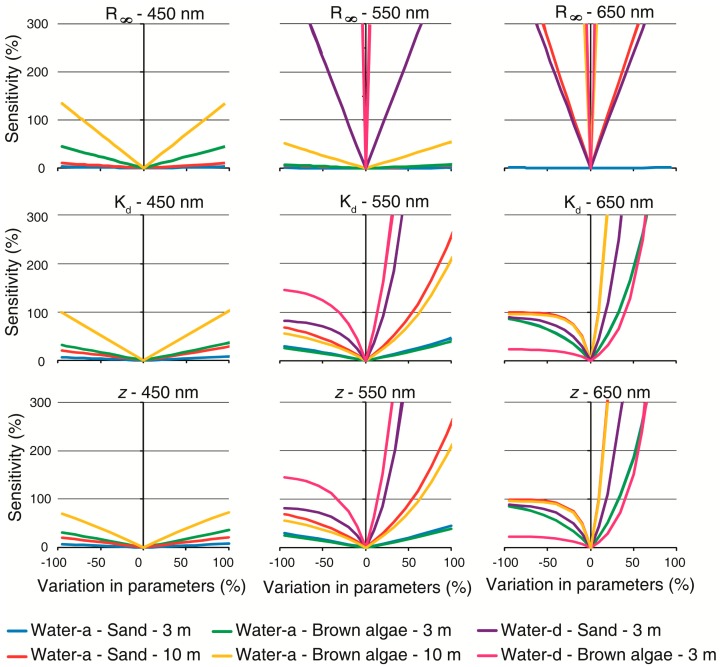
Sensitivity analyses for parameters of M94: *R_∞_*, *K_d_* and *z*. Values correspond to sensitivity (in %) defined in Equation (14). Results are arranged by parameter and wavelength (450, 550 and 650 nm).

**Figure 8.**
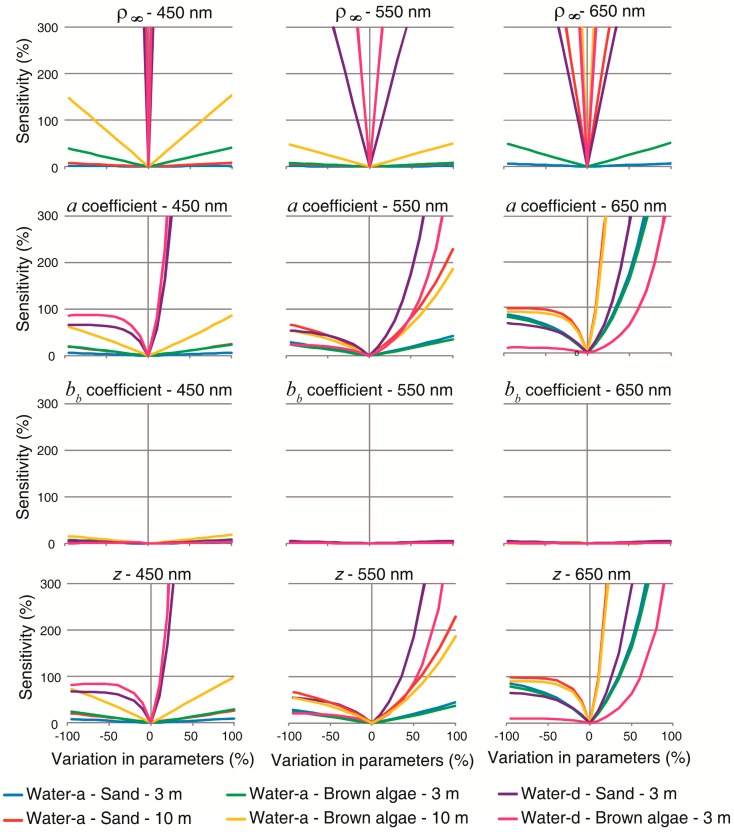
Sensitivity analyses for parameters of L99: ρ*_∞_*, *a*, *b_b_* and *z*. Values correspond to sensitivity (in %) defined in Equation (14). Results are arranged by parameter and wavelength (450, 550 and 650 nm).

Both algorithms showed similar sensitivity to variation in individual parameters (Figures 7 and 8). L99 exhibited a similar response pattern to *a* coefficient than M94 to the *K_d_* because *b_b_* was so low than its contribution to water attenuation was negligible in comparison with *a*. At 450 and 550 nm, models sensitivity showed a linear response in all of the parameters in the clearest water and shallowest situations.

This response was symmetric at 450 nm considering either underestimation or overestimation in the parameters, and as length path increased, the algorithms exhibited increased sensitivity. In situations where substrates were located below a smaller path length in water (small *K_d_* or *a*, and *z*) models seemed insensitive to *R*_∞_ or ρ_∞_. In contrast to deep water reflectance, attenuation and depth, e.g., the parameters acting in the exponential term, had an asymmetrical response according to underestimation or overestimation for long path length (high attenuation and/or *z*). This occurred because an increase on *K_d_*, *a* or *z* reduces exponentially the denominator in Equations (7) and (10). Considering variations in either of the parameters, the algorithms showed an exponential behavior in their sensitivity to overestimations in the most turbid water (water-d). For example, overestimations lower than 50% impacts the retrievals by more than 300%. At 650 nm, not one situation showed a linear behavior in sensitivity to *K_d_* and *z*. The response patterns were similar than at 550 nm; however at 650 nm sensitivity was higher. Both algorithms were insensitive to *R*_∞_ or ρ_∞_ variations at 650 nm in the clear and shallowest water, while model sensitivity increased non linearly in deeper and more turbid waters.

Comparing the most reflective bottom (sand) with a lesser reflective bottom (brown algae), algorithms was less sensitive for sand. In the clearest and shallowest situation (water-a, 3 m), both methods seemed to be robust. However, sensitivity was higher when water path length increased. As the bottom contribution becomes larger, the effect of water column is less important reducing sensitivity. Analogously, according OAC concentration or depth increase, contribution of bottom to surface reflectance decreases while water column contribution increases. It means that small errors in estimating all parameters (attenuation, depth and deep water reflectance) can translate into large ρ*_b_* errors.

Although errors in the modeling could be a large factor for accurate retrievals, some conclusions can be drawn from the analysis. In general uncertainties are higher when optical path length is higher and sensitivity associated with both algorithms is also higher in this case. Depending on depth and *K_d_*, it is not always possible to retrieve a bottom signal or the retrieved signal might be subject to a great degree of uncertainty. This means that it is essential to know the environment under study to evaluate if the algorithm is properly recovering the bottom reflectance or if it is creating an artifact. Validation of the water column correction is desirable when using *in situ* bottom reflectance; however, it can be difficult to measure in the field. In addition, measurements of the bottom reflectance used to be performed very close to the target to minimize water interference, and the resulted IFOV is very small. Considering that the substrate in coral reefs can be highly heterogeneous, punctual measurements are not representative of larger areas (1–900 m^2^ depending on the configuration of the remote sensor); therefore, an understanding of light behavior in water as well as of the study area, such as the water column characteristics and real bottom reflectance at some locations, are required to be able to interpret such measurements properly.

**Figure 9.**
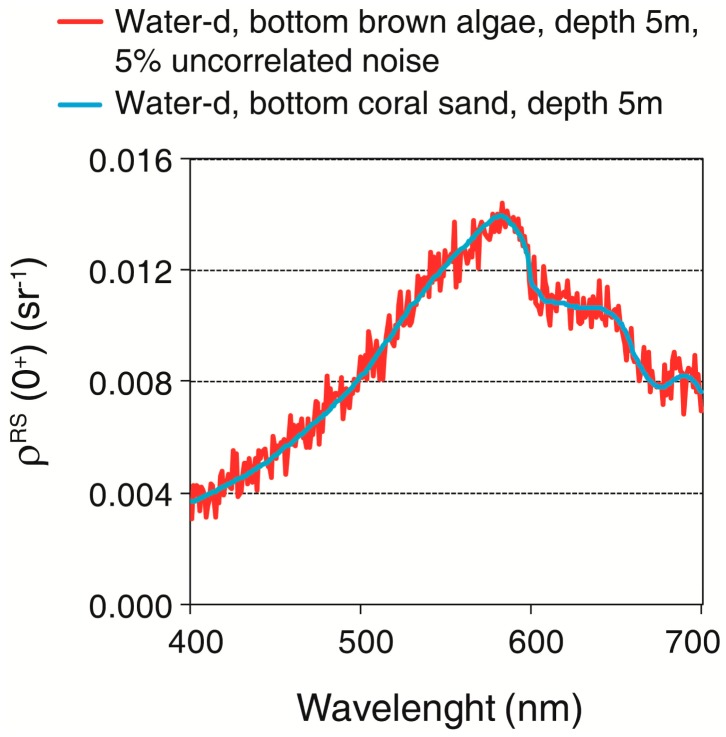
Simulated remote sensing reflectance (sr^−1^) above water as a function of wavelength (nm) in shallow waters (5 m depth) with chl-*a* = 9 μg·L^−1^, a_CDOM_(440) = 0.3, suspended particles Type I = 10 mg·L^−1^, suspended particles Type II = 1 mg·L^−1^ and a_d_(440) = 0.5. The blue curve corresponds to brown algae substrate, while the red one represents coral sand substrate including 5% of uncorrelated noise.

Using the CRISTAL method, each of the 48 ρ*^RS^*(0^+^) spectra was associated with the others in the spectral library whose SAM value was the minimum, and both spectra as considered as corresponding to the same bottom. Therefore, the result of this method was a categorical classification. The accuracy obtained was: 81%, for brown algae, 88% for green algae and 94% for sand. There was some confusion between the three classes (Table 3) that occurred in water-c and -d, which were optical complex Case-2 waters. This technique showed a satisfactory result, and therefore has the potential to be used to correct images. Nonetheless, several considerations are important. First, when the *K_d_* and depth increase, the same spectra should be obtained at the surface for different bottoms. As example, in Figure 9 two ρ*^RS^*(0^+^) spectra were modeled in water-d at 5 m depth, for a sand bottom and the other for brown algae, with 5% uncorrelated noise for the latter. If the noisy pattern of the brown algae spectrum is neglected, both curves showed the same shape. Second, the technique requires measurements of the bottom reflectance of all of the bottoms present in the area and in all combinations in which they might occur. If these inputs do not represent all of the variety present in the field, the technique will not retrieve the real type of bottom in a pixel. In this work, we used the exact same pure substrates that we wanted to retrieve, which means that we used the most favorable conditions in constructing our spectral library. The confusion could be higher if different combinations of substrates are used.

While the three methods tested here can be used to correct the water column effect, it is not simple to compare the performance of M94 and L99 with CRISTAL model because the output is different. While the first two retrieve a numerical value of the bottom reflectance without the effect of the water column, the CRISTAL method produces a categorical result. When applied to an image, the M94 and L99 methods will result in a matrix with continuous values in each spectral band, whereas the CRISTAL method will produce a map with classes of bottoms. The choice of the method depends on different factors, such as the objective of the work, available input data, type of data (multi or hyperspectral), time of processing, *etc*. Whatever the chosen method, it must have some *in situ* data to perform the water column correction.

#### 4.2. Application and Comparison of Selected Methods: Remote Sensing Data

**Figure 10.**
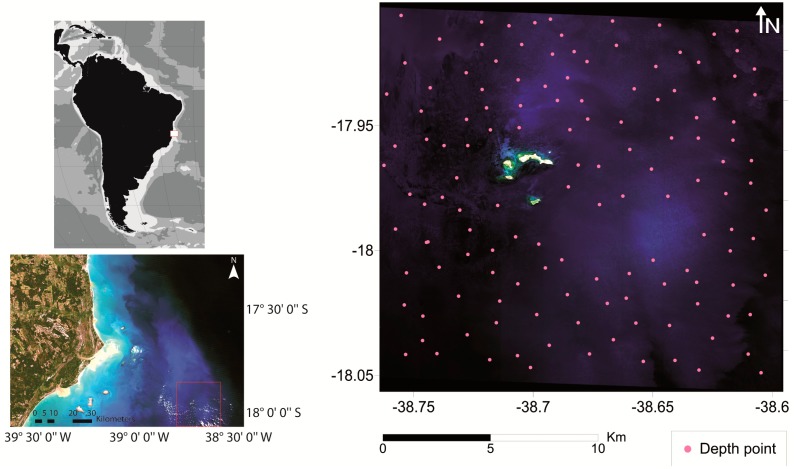
Quasi-true color composition (R: 659 nm; G: 546 nm; B: 478 nm) of a portion of the Abrolhos Coral Reef Bank, Brazil, around the archipelago, captured by WV02 sensor in 14 February 2012. Pink dots show distribution of depth points in the area (**Right**); Red square in image Landsat TM-5 (R: 660 nm; G: 560 nm; B: 458 nm) captured in 29 May 2006 shows location of study area respect to coast (**Lower left**); Location of study area in South America (**Upper left**).

In this section, we intend to correct some spectra extracted from a WV02 scene using M94 and L99. The CRISTAL method was not used because we did not have actual bottom reflectances, which are required to simulate the spectral library. The WV02 image was captured on 14 February 2012 between coordinates 17°54′9.38”–18°3′22.71”S/38°35′43.25”–38°45′38.17”W and corresponds to a portion of the Abrolhos Coral Reef Bank, Brazil (Figure 10). In this area depths vary between 2 and approximately 25 m and some reef structures show a typical mushroom shape whose tops have a diameter between 20 and 300 m [86].

The WV02 sensor collects radiance in 8 spectral bands centered at 427, 478, 546, 608, 659, 724, 831, 908 nm and its nominal spatial resolution is 2 m. The image was atmospherically corrected using the package ATCOR2 available in PCI Geomatica v.10.3.2, and visibility was set at 43 km. The scene was also corrected for sunglint effects [22]. We obtained bathymetric information for 117 points inside the scene, provided by the Brazilian Navy. These points were homogeneously distributed in the scene and depths were corrected to a tidal height at the time of the imagery. Spectral curves of the surface reflectance (adimensional) (ρ*_w_*(0^+^)) were extracted in the same pixels where we had depth data. Several samples in deep areas were carefully selected, and mean values were calculated for each band. These values were used as input to both algorithms to represent the deep water reflectances.

We collected hyperspectral profiles from 349.6 to 802.6 nm of *E_d_*, *L_u_* and scattering in 700 nm quasi-concomitant with the imagery (between 27 February 2012 and 29 February 2012). Profiles were registered between the surface and bottom in the deepest areas of the scene (20–25 m) at different times of the day and for 2–3 replicates of the profile. *E_d_* (μW·cm^−2^·nm^−1^) and *L_u_* (μW·cm^−2^·nm^−1^·sr^−1^) measurements were obtained using HyperOCR sensors connected to a Satlantic Profiler II.

The Satlantic Profiler II also has an ECO BB3 sensor that measured the backscattering coefficient in the water column. All data collected with the Satlantic Profiler II were processed using Prosoft 7.7.16 software to obtain: *K_d_* profiles; ρ*^RS^* at 440 and 555 nm; and *b_bp_* at 700 nm. The hyperspectral *K_d_* along the water column was averaged for each profile excluding measurements in the 5 first meters because the *E_d_* showed a noisy pattern, mainly caused by waves and bubbles. Then, a mean *K_d_*(λ) value between all profiles was obtained. Additionally, water samples were collected in field between 13 February 2012and 3 March 2012 and filtered following NASA protocol [87] to obtain the chl-*a* concentration and absorption coefficients of the detritus (*a_d_*) and phytoplankton (*a_phyto_*). We used WASI software in the inverse manner to retrieve *a_CDOM_*. In this sense, the *K_d_* spectrum was used as an input and chl-*a* concentration was fixed at 0.48 mg·m^−3^ according to our estimates in the field. At last, *a* was obtained as the sum of *a_CDOM_*, *a_d_*, *a_phy_* and *a_w_* [88]. *b_bp_* was derived through the QAA method [31] using the ρ*^RS^* at 440 and 550 nm registered with the Satlantic Profiler II. It was validated using *b_bp_* at 700 nm measured *in situ. b_b_* was estimated as the sum of *b_bp_* and *b_bw_* [89]. The hyperspectral data of *K_d_*, *b_b_* and *a* were integrated over the spectral bands of WV02 up to 700 nm.

We corrected 117 spectra from the WV02 image for which we had depth information. M94 and L99 were applied using Equations (7) and (10) respectively. The inputs for M94 were *z*, *K_d_*, *ρ_w_*(0^−^) and ρ_∞_(0^−^). The above surface reflectances were converted to below water reflectances using Equation (11). The inputs for L99 were *z*, *a*, *b_b_*, ρ*^RS^*(0^−^) and $\rho_{\infty }^{\text{RS}}(0^{-})$. The above-water surface reflectances obtained from atmospheric corrections were divided by π to convert them to remote sensing reflectances, with the surface considered as Lambertian, and converted to below-water reflectances. Above remote sensing reflectances were also converted to below-water reflectances (Equation (11)).

**Figure 11.**
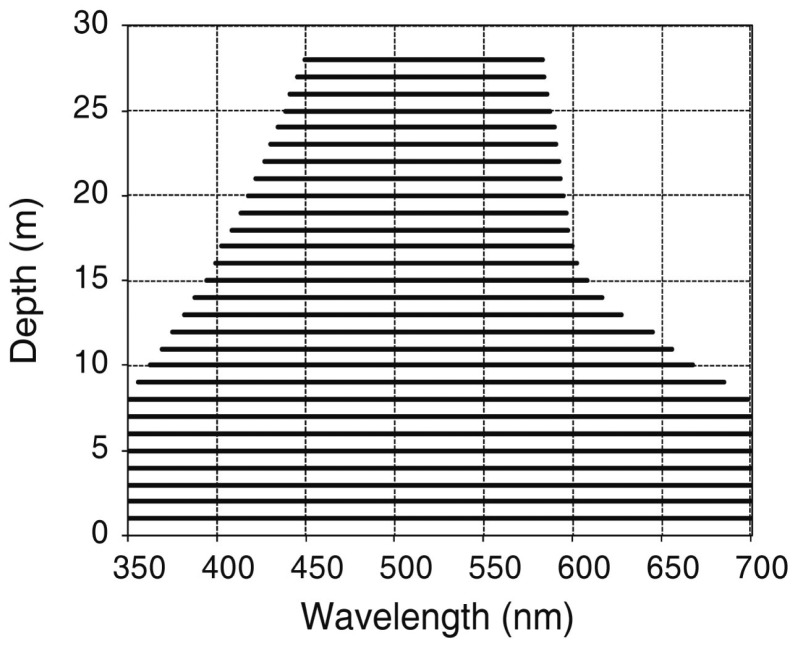
Maximum range of wavelength in which a substrate (composed by coral sand, green and brown algae) located at different depths can be detected with remote sensing techniques.

To analyze the bottom retrieval for both methods, we first excluded values for which the algorithm was invalid (0 > ρ*_b retrieved_* > 1). Then, we excluded values where typically no bottom contribution to surface reflectance was expected. The bottom contribution to surface reflectance depends on bottom reflectance itself. Since we did not have real ρ*_b_* in our area, others simulations were performed in the WASI software considering a standard bottom constituted by 1/3 coral sand, 1/3 brown algae and 1/3 green algae. The bottom was simulated at 28 different depths between 1 and 28 m in a water medium with the same parameters estimated for the day of WV02 imagery. The bottom contribution was estimated through Equations (8) and (13), and both algorithms were slightly different in their estimation. For each depth, we calculated the mean range of wavelengths for which the bottom contribution at the surface was received. Figure 11 shows the shrinkage in wavelength range according to depth increase. The water column characteristics in our study area were similar to the water-b simulated in Section 4.1. Hence, we also excluded values where exponential behavior was expected according to our previous results.

ρ*_b retrieved_* by L99 showed a higher percentage of invalid values at all depths. For example, between 9 and 11 m depth M94 retrieved 0%–16% invalid values, whereas L99 failed between 9% and 88%. Considering 13–16 m depth, the M94 retrieved invalid values in 25%–58% of the cases, whereas Lee's model retrieved 24%–100% invalid results. Therefore, for the selected imagery, the M94 seemed to show a better performance than the L99.

**Figure 12.**
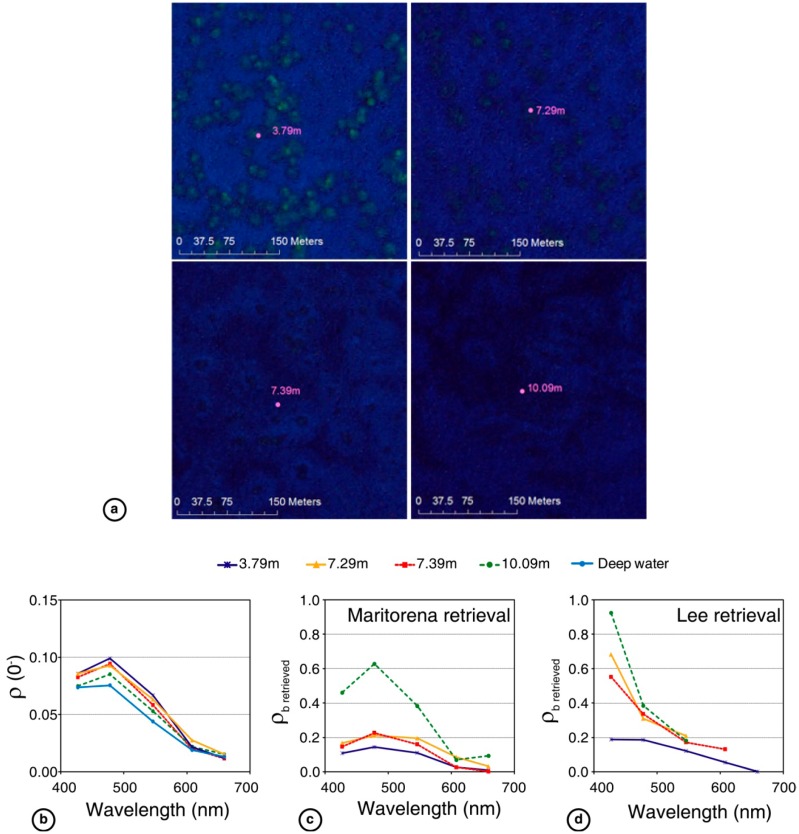
(**a**) Zoom in different portions of WV02 image in quasi-true color (R: 659 nm; G: 546 nm; B: 478 nm). All images have exactly the same contrast and are in the same scale. Pink circles show location of depth points and their values are indicated; (**b**) Reflectance below water *versus* wavelength (nm) captured by WV02 sensor above the four points located in (a) and above deep water; (**c,d**) Bottom reflectance below water *versus* wavelength (nm) retrieved by M94 and L99, respectively.

Although all surface spectra in the shallow water seemed similar, they were influenced by the bottom because deep water showed a lower spectrum than shallow water. As the bottom depth increased, the surface spectrum had reduced magnitude and approximated the deep water spectrum. However, similar spectra for the surface water when the bottom was located at different depths can be explained by the differences in bottom reflectance. Indeed, depth points were clearly located above areas with different bottom characteristics (Figure 12a). These differences can be observed as slight discrepancies in ρ(0^−^) spectra (Figure 12b). If bottoms located at very distinct depths showed uniform reflectance patterns, it meant that they were not of the same kind of bottom and exemplified the importance of applying water column corrections. After M94 was applied, the ρ*_b retrieved_* of these spectra showed a divergence, not only in their shape but in their magnitude (Figure 12b). Note that substrates at similar depths (at approximately 7 m) exhibited similar magnitudes in ρ*_b retrieved_*. Moreover, substrates at 3.79 and 7.29 m in Figure 12 are located above the top of the reefs and are expected to have similar composition in a biological community dominated by corals, turf and crustose algae [90–92]. Likewise, according to a visual inspection, points at 7.39 and 10.09 m are expected to be composed of the same type of substrates: sand and macroalgae. In fact, retrieved spectra in each pair of locations showed the same shape, which suggested the same type of bottom. Nonetheless, it seems that the algorithm can properly retrieve the shape of a spectrum in some bands but fail to retrieve its correct magnitude. ρ*_b retrieved_* through Lee's algorithm presented a peak in the shortest wavelength (427 nm) followed by an abrupt decay toward the longer wavelengths (Figure 12b). Only bottoms at the shallowest points exhibited a similar shape as the M94 retrieval. In this case, an increase in ρ*_b retrieved_* was also observed according to bottom depth increase.

Several uncertainty sources may cause increasingly large biases in retrieved bottom reflectance as depth increases. For example, *K_d_*, *b_b_*, and *a* were not estimated exactly at the time of the imagery, and this can introduce errors in results. As we observed in the sensitivity analyses in Section 4.1, uncertainties in these inputs can have an important impact in retrievals and they can be related to the depth. Besides that, all reflectance models are based on the exponential decay of light. In the first meters of the water column, the *E_d_* profile showed a noisy pattern because of environmental factors such as waves, bubbles, OAC stratification, and fluctuations of the surface [93–95]. It means that light could not perfectly decay exponentially, in particular considering shallow depths such as in this analysis. If the light decay is not exactly exponential, the algorithms will tend to retrieve skewed bottom reflectances as the depth increases. The spectral shape of ρ*_b retrieved_* from both algorithms showed maximum values in shorter wavelengths, where the attenuation coefficient was lower. The natural substrates (e.g., sand, algae, corals, mud) do not have this type of reflectance curve, revealing that algorithms failed to retrieve the bottom reflectance below 500 nm. Above 600 nm, there are no differences between shallow and deep waters, indicating that there is no contribution of the bottom in these bands even in the lowest depth.

**Figure 13.**
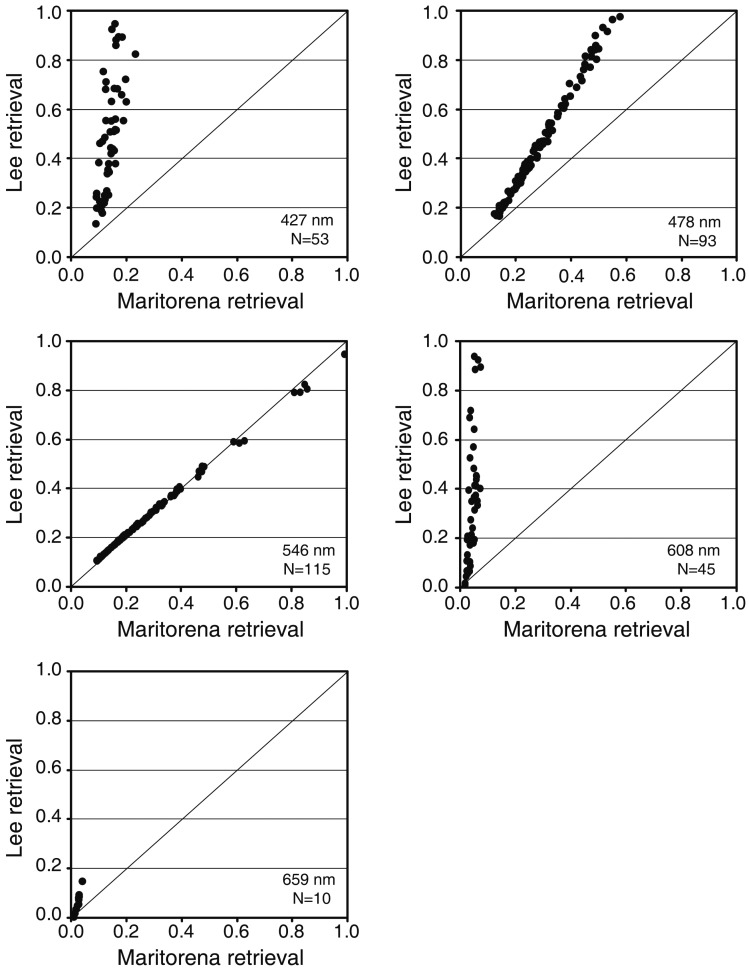
Bottom reflectance retrieved using L99 *versus* M94 retrieval from WV02 image. Each plot corresponds to a different wavelength (427, 478, 546, 608 and 659 nm). Straight lines correspond to proportion 1:1.

Accordingly, the bands between 400 and 600 nm can contribute to the bottom differentiation. In this sense, the WV02 has an advantage over other orbital sensors with high spatial resolution because it has 4 bands inside this interval. The models apparently fail to simulate properly water reflectance at the surface above shallow bottoms and acting attenuation processes appear to be different in nature than in the simulations for models developed until now. Comparing the retrievals by both algorithms, an overestimation by Lee's algorithm can be observed (Figure 13).

However, both retrieved exactly the same value at 546 nm in all depths, which was precisely in the band where there was the lowest percentage of invalid values. The overestimation by L99 could correspond to an exponential behavior. Notice that N is higher at 546 nm because the invalid values were removed from the shorter and longer wavelengths. To correct simulated spectra in an orbital multispectral image, M94 was more efficient. This method is also advantageous because it uses a lower quantity of inputs. Methods requiring a large number of input parameters may produce worse results because of uncertainties in estimating each parameter that propagate to the results. Goodman *et al.* [72] noted that pre-processing steps, such as atmospheric and sunglint corrections, are important because they could have a large impact on the results of the L99 method, especially when large amounts of cross-track sunglint is present in an image. To obtain better results, these authors also suggested using *Q* in the equation for bottom reflectance (*Q* = *E_u_*/*L_u_*, the ratio of upwelling irradiance to upwelling radiance at nadir) instead of using π.

### 5. Discussion

In recent years, the number of studies of coral reef ecosystems using remote sensing has increased substantially. However, they require the use of correction algorithms for the effects produced by the water column to compensate for those caused by the depth and optically active constituents. The application of an appropriate algorithm for correcting the water column effects and accurately analyzing the input data, along with the development of new algorithms, will result in radiometric data with minimal water column effects that increase the accuracy of the mapping of reef ecosystems. Water column corrections minimize the confusing effects caused by different depths in scenes but do not eliminate this effect. At long wavelengths and depths, it is difficult to retrieve the bottom reflectance because of large light absorption by the water molecules. In the following, we further discuss the main algorithms available for water column correction, emphasizing advantages and drawbacks.

Lyzenga's algorithm [35,36] almost does not require the field data of the water column, which are difficult to obtain, especially in lesser studied reefs. For this reason and because it was one of the first algorithms, it is by far the most applied. However, it only can be applied in clear waters. The result is a relation between bands that can be useful for bottom type mapping but not for other purposes when reflectance spectra are required, also impeding its validation. Spitzer and Dirks [54] produced applications of the Lyzenga algorithm for LANDSAT and SPOT satellites that are better for some situations depending on depth and substrate type. However, the limitations are the same as with Lyzenga's method. In cases such as MSS/LANDSAT and SPOT-XS sensors, this could mean reduced results to a unique band. To isolate the index between two spectral bands, Conger *et al.* [39] proposed a helpful solution that can only be applied in bands located in short wavelengths, because of the deeper penetration of light in this region. Analogously, another possibility to be explored in particular areas could be using different points across a scene with the same depth and over the same substrate. Using differences in trajectory extensions caused by the viewing configuration, *K_d_* could be estimated.

Tassan [37] and Sagawa *et al.*'s algorithm [38] gave alternatives to Lyzenga's algorithm to overcome the requirement of clear water. While Tassan's algorithm produces a band-relation index, Sagawa *et al.*'s algorithm produces a derivation of bottom reflectance in each band; however, bathymetric information is required in the entire scene. The previous methods allow *K_d_* to be obtained from the same image and do not need *in situ* measurements in the water column.

Despite their limitations, the methods discussed so far could be good starting points for coral reefs that are being studied by remote sensing. Conversely, Bierwirth *et al.*'s algorithm [34] only needs *K_d_* measured in field and does not require a bathymetric map. The result is a derivation of real reflectance that is different from the previously mentioned methods because it is band independent. Bertels *et al.*'s approach [60] does not correct water column effect but is an alternative that can also be useful in particular cases in initial studies. The empirical algorithm from Gordon and Brown [55] is based on Monte Carlo simulations and requires some knowledge of the *b* and *c* parameters in the water column, but they are not input parameters. In comparison to empirical algorithms, analytical and semi-analytical algorithms use *in situ* measurements as inputs. Within semi-analytical methods, M94 [56] proposed a simple method that uses a limited number of measured parameters as inputs and the shape of retrieved bottom reflectance adjust well with measured ones when the study area is located in waters with low OAC concentration. This method tends to underestimate this output parameter. Purkis and Pasterkamp [57] introduced a multiplicative factor that accounts for the effects on the water surface because the bottom reflectance resulting from the application of this model is evaluated above the surface. L99 [58] and Yang *et al.*'s algorithm [59] require a greater number of *in situ* measurements. The first one takes into account several processes that occur in the water column to constitute a realistic model and observation geometry. The second one [59] appears to be robust in retrieving the reflectance spectra and allows for the incorporation of vertical and horizontal heterogeneity associated with depth; therefore, it is a more realistic algorithm. The algorithm that requires the most quantity of inputs parameters is its counterpart, and for each layer of the water column, a thorough understanding of the environment is required. It can also be computationally heavy.

Different methods have been developed for passive sensors for both multi and hyperspectral resolutions. Simpler correction methods have been developed for multi-spectral data of a few bands and consider clear water with vertical and horizontal homogeneity. For this reason, image correction fails when it is applied to deep or turbid waters. However, these methods have great potential because they only require a small amount of *in situ* data and may be useful in some regions. On the other hand, newer methods allow considering higher environmental variability in water column related with depth. Although coral ecosystems are in clear water environments, horizontal heterogeneities in water transparency can often occur. This means that the light attenuation may have some degree of spatial variability, especially in shallower areas. Thus, sometimes using a single attenuation coefficient in an entire scene, often obtained for deep water, can be inappropriate. For this reason, the assumption of horizontal homogeneity is a limitation for all of the models. New methods for water column correction should consider this variability. A viable alternative would be to break the image into homogeneous areas for the application of different attenuation coefficients or different water correction methods. Another input required in most methods is the depth. Hereby, the availability of accurate bathymetry that is normalized by the tide height for the time of the image collection and with a spatial resolution match is a requirement to obtain a satisfactory result. Few methods consider inelastic scattering (Raman) and none take into account the fluorescence of the phytoplankton and CDOM. However, in some situations, these processes may have important contributions, and their inclusion in the models can improve the fit.

Optimization/matching algorithms start with a hyperspectral surface reflectance and are inverted or matched with a spectral library to simultaneously obtain depth, OAC in water and bottom type in each pixel. For this reason, this group of models is capable to account for horizontal heterogeneity in the water. The different approaches mainly differ in the type of creation of the spectral library, matching algorithms and the type of composition of bottom. The simplest method in this group is Louchard *et al.*'s approach [6], which only retrieves bottom types and bathymetry for known parameters. Because a wide variety of bottom reflectance spectra measured *in situ* is used in creation of spectral library, the model was capable of retrieving several substrate classes. The CRISTAL method requires a significant time to construct the spectral library but has the following: it offers a large range of bottom types to be retrieved, and no assumptions about the water column characteristics are performed. Precisely because of that, ambiguous results can be generated by this approach in turbid or deeper waters. To retrieve the real bottom reflectance, these methods are strongly dependent on bottom reflectance spectra used as input to generate the spectral library. BRUCE and SAMBUCA are inversion methods derived on the algorithm by L99 [58]. They only use two or three substrate types because they were initially applied in areas with a small diversity of the bottom, and after their application, the proportion of each bottom type in every pixel is retrieved. The ALUT algorithm proposes to divide the range of variation of the parameters in an efficient way. Therefore it offers a fast application that promises accurate results. Whatever the method used in this group, an error in an estimating any parameter could lead to errors in the others.

After carefully investigating each method, the question that inevitably arises is what is the best method? The answer is not simple and each method has its advantages and limitations and can work properly in certain environments or potentially fail. For example, some methods assume homogeneity in the distribution of constituents of the water column while others can consider heterogeneities in the water between pixels. But inputs and outputs also differ between algorithms. Benefits and disadvantages of each technique have been discussed when describing individual algorithms, when commenting on the comparative analysis made (previous and those accomplished as part of the present study), and summarized at the beginning of the discussion. Hence, the best correction model to be chosen depends on the environment and sensor characteristics, mapping purposes and available *in situ* data. When a new work is being planned, acquisition of remote sensing images can be planned and field data collection can be designed. In this situation, water column corrections could be chosen in advance, for which a critical comparison of the performance of methods would be desired. Nevertheless, in an attempt to produce this comparison, we found certain restrictions. Most of the algorithms did not provide a validation of their retrieval or each author validated their method in a different way. Some of them evaluated the adjustment between the simulated and measured bottom reflectance, using different statistics parameters (R^2^, R, RMS) or by visual comparison. Others scarcely inspected the scene visually after application of the water column correction algorithm, whereas others did not show whether the water column correction improved the mapping accuracy. Comparisons between the map accuracy can also be subjective because it depends on a number of classes, sensor configurations, classification algorithms and environmental characteristics of the area, among others. For this reason, works such as Dekker *et al.*'s [75] that produce an objective inter-comparison between different methods are required for the process of selecting an appropriate method and are strongly encouraged.

This work also provides a simple comparison between methods based in radiative transfer models. Their application showed that the algorithm performance varied with depth, OAC concentration and type of bottom. Even considering the homogeneous water column in an image, different performance of water column correction algorithms is expected between pixels according to the substrate and depth. It is an aspect that has to be included when mapping accuracy is developed. For example, validation points should be distributed in an area to be representative of all conditions found in a scene. Using simulated spectra, our results showed that in clear waters and depth lower than 10 m it is possible to retrieve moderately accurate bottom reflectance, and they are consistent with Dekker *et al.* [75]. When retrieving bottom reflectance from the WV02 image, results seemed to be more degraded. However, the lack of actual bottom reflectance measured *in situ* impeded a quantitative estimation of their accuracy. Although L99 inversion scheme [58] was previously applied with success to retrieve the water coefficients, it was developed considering a homogeneous reflective substrate. Its efficiency was lower for retrieving bottom reflectance. Even when M94 failed to retrieve the bottom at longer wavelengths, it showed a higher performance and can be easily applicable with just a few inputs. Algorithms are still not capable of completely separating the water from the bottom reflectance, and because the performance of models is depth dependent, they do not yield accurate bottom reflectances and do not eliminate completely the effect of depth.

With more *in situ* data that are known from the water column, it is expected that a more realistic situation could be simulated and better results would be obtained. In the ideal condition, it would be desirable to know all of the IOPs from the water column, depth, and atmospheric conditions across the entire scene at the time of the image acquisition together with a significant quantity of points of bottom reflectance spectra to validate the results. Thus, what is the advantage in using remote sensing if so much data are required for reliable and accurate results? First, remote sensing offers an extent of spatial and simultaneous data collection that cannot be achieved with other approaches. Second, an initial effort is required at the beginning when a new area is explored; areas that are more well-known and higher quantities of available data increase the reliability of a model and require fewer ground truth as model inputs. An endeavor to determine the water characteristics (e.g., *K_d_*, IOPs, OAC concentrations) in an area across time should be rewarded with the successful application of water column corrections. Once these parameters are known, they could be used in other studies at the same season of year if they do not coincide with extreme weather or biological events, such as occurrence of hurricanes, phytoplankton bloom events or spawning events.

Coral reefs active remote sensing is a complement to passive remote sensing multispectral and hyperspectral data for bottom mapping in coral reefs because fluorescence signals from LIDAR measurements can be used to estimate the water column characteristics, bathymetry and habitat complexity [96]. Fluorescence signals also provide information to differentiate between dead and healthy coral. In addition to retrieving bathymetry, active remote sensing offers the opportunity to estimate different AOPs and IOPs from fluorescence and polarization measurements from airborne platforms [97,98].

### 6. Conclusions

Water column correction is a required step extracting information from shallow bottoms using remote sensing optical images. Among all the methods developed to date, none are generally capable of correcting for the water column effect properly in the entire visible spectrum. Even in the best conditions, it is not possible to be completely depth independent because uncertainties depend on the wavelength, bottom depth and type of bottom. In all cases, some knowledge of the water column constituents, depth, and spectral behavior of substrate is required as input during the execution of a method or to evaluate its performance after application.

The first proposed algorithms were the simplest and easy to apply as a band ratio. Their limitations notwithstanding, they are still the most frequently used algorithms. More complex algebraic algorithms have been developed to estimate the reflectance in shallow environments that require more field data. They are the only methods capable of estimating bottom reflectance. For this reason, improvements and validations of this group of algorithms should be encouraged. Most of the recent algorithms have been based on matching pixel spectra with simulated spectra from a library or inversion algorithms. While these algorithms produce satisfactory results, their output is a categorical map and their performance is dependent on realistic bottom reflectance data sets. Basically, the choice of method to apply is dictated by the available input data and the desired outcome in terms of output variable and accuracy based on the scientific study envisioned.

## Figures and Tables

**Table 1.** Summary of models reviewed in this paper considering methodological approach, data spectral resolution, data required, model results and other observations.

**Reference**	**Approach**	**Spectral Resolution**	**Required Input and Main Equations**	**Assumptions/Applicability**	**Algorithm Output**
Lyzenga [35]	Band combination	Multispectral	*L_TOA_*, *L_TOA,_*_∞_ in at least, in two bands. Pixels samples of the same substrate (homogeneous) depth known, occurring in a wide range of depths. ${\text{Index}}_{ij}=\ln\left(L_{\text{TOA},i}-L_{\text{TOA},\infty ,i}\right)-\left[\left(\frac{K_{d,i}}{K_{d,j}}\right)\ln\left(L_{\text{TOA},j}-L_{\text{TOA},\infty ,j}\right)\right]$	Water column vertically and horizontally homogeneous; small variability in bottom reflectance for the same type of substrate. Applicable in high transparency waters. The model cannot be applied to very shallow waters.	Composition of bands *i*, *j*
Spitzer and Dirks [54]	Band combination	Multispectral (MSSTM/LANDSAT, HRV/SPOT)	*L_TOA_*, *L_TOA,_*_∞_ in at least two spectral bands in the visible region, obtained by MSSTM/LANDSAT or HRV/SPOT sensors. $\begin{array}{c} {\text{Index}}_{b1}=\ln\left(L_{\text{TOA},2}-L_{\text{TOA},\infty ,2}\right)-0.3\ln\left(L_{\text{TOA},3}-L_{\text{TOA},\infty ,3}\right)+h_{b1}\\ Index_{b2}=\ln\left(L_{\text{TOA},1}-L_{\text{TOA},\infty ,1}\right)+\ln\left(L_{\text{TOA},2}-L_{\text{TOA},\infty ,2}\right)-0.6\ln\left(L_{\text{TOA},3}-L_{\text{TOA},\infty ,3}\right)+h_{b2}\\ Index_{b3}=\ln\left(L_{\text{TOA},2}-L_{\text{TOA},\infty ,2}\right)-1.05\ln\left(L_{\text{TOA},1}-L_{\text{TOA},\infty ,1}\right)+h_{b3} \end{array}$	Same assumptions of Lyzenga's model. Applicable only for LANDSAT and SPOT satellites.	Composition of two or three bands
Tassan [37]	Band combination	Multispectral	*L_TOA_*, *L_TOA,_*_∞_ in at least two spectral bands. Pixels samples (depth known) of two homogeneous substrates: high and low bottom albedo, occurring in a wide range of depths. $\begin{array}{c} {X^{\prime }}_{i}=\ln\left[L_{\text{TOA},i}-L_{\text{TOA},\infty ,i}\right]\\ {X^{\prime }}_{ij}={X^{\prime }}_{i}-\left[\left(K_{d,i}/K_{d,j})(\text{low albedo}\right)\right]{X^{\prime }}_{j}\\ {X^{\prime }}_{ij}={X^{\prime }}_{i}-\left[\left(K_{d,i}/K_{d,j})(\text{high albedo}\right)\right]{X^{\prime }}_{j} \end{array}$	Can be able to be applied in scenes with turbidity gradients between shallow and optically deep waters. Assumes vertically homogeneity. The application of this method is sequential.	Composition of bands *i*, *j*
Sagawa *et al.* [38]	Band combination	Multi and hyperspectral	*L_TOA_*,*_I_*; *L_TOA,_*_∞_*_,I_*; *k_i_*, *q* and *z* $Index_{i}=\frac{\left(L_{\text{TOA},i}-L_{\text{TOA},\infty ,i}\right)}{e^{\left(-K_{d}qz\right)}}=m_{i}\rho_{b,i}$	Vertical and horizontal homogeneity. Can be applied in environments with low water transparency. Accuracy in bathymetric map is important to obtain a reliable result.	Index proportional to ρ*_b,i_*
Conger *et al.* [39]	Band combination	Multi and yperspectral	*L_TOA,i_* pixel samples of the same homogeneous substrate, depth known, *L_TOA,_*_∞_*_,i_*	Assumes vertical and horizontal homogeneity and mall albedo variability of the substrate samples. his method is not effective in the red band.	Pseudo-color band, epth independent
Gordon and rown [55]	Model-Based lgebraic	Multi and hyperspectral	*R_i_*, *c, b R_i_*(0^+^) = *R*_1_ + [ρ*_b_R*_2_/(1 − *s*ρ*_b_*)]	Vertical and horizontal homogeneity. Empirical estimation of model parameters.	ρ*_b_*
Maritorena *et al.* [56]	Model-Based Algebraic	Multi and hyperspectral	*R_w_*, *R*_∞_, *z* in each pixel of the scene, *K_d_ R_w_* = *R*_∞_ + (ρ*b* − *R*_∞_)*e*^[−2^*^K_^d^_z^*^]^	Assumes a vertical and horizontal homogeneity and high water transparency.	ρ*_b_*
Bierwirth *et al.* [34]	Model-Based Algebraic	Multispectral	ρ*^RS^*, *K^d^* two or more spectral bands are required, *N* corresponds to number of spectral bands used $\begin{array}{c} \rho_{B}^{RS}=\rho^{RS}(0^{-})e^{\left(2K_{d}Z_{\alpha }\right)}\\ Z_{\alpha }=\overset{N}{\underset{i=1}{\text{∑}}}\frac{\ln\rho^{RS}(0^{-})}{\left(-2K_{d}N\right)}\\ \rho_{B}^{RS(1/2K_{d})}=\rho_{b}^{RS(1/2K_{d})}e^{\Delta z} \end{array}$	Model must be applied in clear water environments. Bathymetric map can be combined with model results and an image with bottom reflectance and depth structure is obtained.	Derivation of the real bottom reflectance.
Purkis and Pasterkamp [57]	Model-Based Algebraic	Multi and hyperspectral	$\begin{array}{c} \rho^{RS}\left(0^{+}\right),\rho_{\infty }^{RS}\left(0^{+}\right),K_{d},z\\ \rho_{b}^{RS}=\frac{\frac{1}{0.54}\rho^{RS}\left(0^{+}\right)-\left(1-e^{-2K_{d}z}\right)\rho_{\infty }^{RS}\left(0^{+}\right)}{e^{-2K_{d}z}} \end{array}$	Water-leaving reflectance does not need previous correction for sea-air interface. Accurate bathymetric data are required. Model must be applied in clear water environments.	$\rho_{b}^{RS}$
Lee *et al.* [58]	Model-Based Algebraic	Multi and hyperspectral	ρ*^RS^*, *θ_v_*, *θ_w_*, *b_b_*, *α*, *z* in each pixel of the scene $\begin{array}{r} \rho^{RS}(0^{-})=\rho_{\infty }^{RS}(0^{-})\left(1-e^{\left[-\left(\frac{1}{\cos\theta_{s}}+\frac{D_{u}^{c}}{\cos\theta_{v}}\right)kz\right]}\right)+\frac{1}{\pi }\rho_{b}e^{\left[-\left(\frac{1}{\cos\theta_{s}}+\frac{D_{u}^{B}}{\cos\theta_{v}}\right)kz\right]}\\ D_{u}^{c}\approx 1,03{\left(1+2,4u\right)}^{0,5};D_{u}^{B}\approx 1,04{\left(1+5,4u\right)}^{0,5}\\ u=\frac{b_{b}}{\left(a+b_{b}\right)};k=a+b_{b} \end{array}$	Assumes vertical and horizontal homogeneity. The model uses detailed information of the optical properties of the water column. Semianalytic model.	ρ*_b_*
Yang *et al.* [59]	Model-Based Algebraic	Multi and hyperspectral	For each water layer: ρ*^RS^*, *K_d_*, *z*, *b_p_*, *f*, β*_w_*(90°, λ_0_), ψ_1_ and ψ_2_ $\begin{array}{l} \rho_{h}^{RS}=\rho^{Rs}(0^{-})-\rho_{\infty }^{RS}(0^{-})\\ \rho_{\infty }^{RS}(0^{-})=Q\left[\frac{e^{\left[-2k_{d}][z-0\right]}-1}{-2K_{d}}\right]{\left[-2\pi \beta_{w}(90^{\circ },\lambda_{0}){\left(\frac{\lambda_{0}}{\lambda }\right)}^{4.32}\left(\cos\psi +\frac{0.835}{3}\cos^{3}\psi \right)-\frac{b_{p}}{2f}\frac{1-f^{2}}{\left(1+f^{2}-2f\cos\psi \right)}\right]}_{\psi_{2}}^{\psi_{1}} \end{array}$	Can be used if the water column is vertically heterogeneous and composed by multiple layers. Within each layer, the optical properties are homogeneous. Analytical model.	$\rho_{b}^{RS}$
Louchard *et al.* [6]	Optimization/Matching	Hyperspectral	Measurements of optical properties, range of depths in area and substrate reflectance occurring in the scene. Data of the geometric conditions of the illumination and image acquisition. Any software that can generate the spectra for the spectral library.	For the first application in an area, it can take long time to generate the spectral library.	Categorical map of bottom type, OAC concentration, z
CRISTAL	Optimization/Matching	Hyperspectral	Measurements of all bottom reflectance occurring in the scene. Any software that can generate the spectra for the spectral library.	For the first application in an area, it can take long time to generate the spectral library.	Categorical map of bottom type, OAC concentration, z
BRUCE	Optimization/Matching	Hyperspectral	ρ*^RS^* substrate reflectance of the main types occurring in the scene ρ*_b_* = *B_sd_R_sd_* + *B_sg_R_sg_* + *B_ba_R_ba_*	Long processing time to generate spectral library. The result is not categorical but a simplification of the main types of substrates occurring in the area. Useful for areas with low diversity.	ρ*_b_*, OAC concentration, z
SAMBUCA	Model-Based Algebraic	Hyperspectral	ρ*^RS^* substrate reflectance of main substrate types occurring in the scene In L99, ρ*_b_* corresponds to: ρ*_b_* = *g_bm,sand_* ρ*_bm_* + (1 − *g_bm,sand_*)ρ*_sand_*	Modification of inversion scheme of L99 but consider that bottom is a linear combination of two types of substrates.	ρ*_b_*, OAC concentration, z
ALUT	Optimization/Matching	Hyperspectral	ρ*^RS^*	ALUT algorithm optimizes the processing time to subdivide the parameters space.	Categorical map of bottom type, OAC concentration, z
Pseudo-Invariant feature (PIF)	Multi-temporal Analysis	Multi and hyperspectral	DN, images of the same sensor and area for different dates perfectly co-registered. Samples of low and high albedo for all dates.	Model assumes the samples are constant in time.	Normalized time series of images
Bertels *et al.* [60]	Geomorphology	Multi and hyperspectral	ρ*^RS^*, Multi and hyperspectral	Useful in reefs where the substrate types and geomorphologic zones are strongly related.	Categorical map of bottom type

**Table 2.** Water characteristics of the four different types of water used to simulate surface reflectance in shallow waters.

Water Type	chl-*a*(μg·L^−1^)	a_CDOM_(440) (m^−1^)	Suspended Particles Type I (mg·L^−1^)	Suspended Particles Type II (mg·L^−1^)	a_d_(440) (m^−1^)	n
Water-a	0.01	0.0017	0.01	0	0	−1
Water-b	1	0.0316	1	0.8	0	−1
Water-c	3	0.15	3.5	2.2	0.2	0
Water-d	9	0.3	10	1	0.5	0

**Table 3.** Confusion matrix obtained for CRISTAL method using the SAM classification algorithm.

		Assigned Class			

		Sand	Green Algae	Brown Algae	
**Real Substrate**	**Sand**	15	0	1	16
	**Green Algae**	1	14	1	16
	**Brown Algae**	1	2	13	16
		17	16	15	48

## Appendix

**Appendix A1.** Acronyms and abbreviations used in this review.

AAHIS	Advanced Airborne Hyperspectral Imaging Sensors
ACRB	Abrolhos Coral Reef Bank
ALUT	Adaptive Look-Up Trees
AOP	Apparent Optical Property
AVIRIS	Airborne Visible/Infrared Imaging Spectrometer
BRUCE	Bottom Reflectance Un-mixing Computation of the Environment model
BSP	Binary Space Partitioning
CASI	Compact Airborne Spectrographic Imager
CDOM	Coloured Dissolved Organic Matter
chl-*a*	Chlorophyll-*a* concentration
CRISTAL	Comprehensive Reflectance Inversion based on Spectrum matching and Table Lookup
DN	Digital Number
HRV	High Resolution Visible
IHS	Intensity-Hue-Saturation
IOP	Inherent Optical Properties
LIDAR	Light Detection And Ranging
LQM	Least Squares Minimum
LSI	Lee Stocking Island, at Bahamas
LUT	Look-Up Tables
MB	Moreton Bay, at Brisbane, Australia
MNF	Minimum Noise Fraction
MSS	Multispectral Scanner
OAC	Optically Active Constituents
OBIA	Object-Based Image Analysis
PCA	Principal Components Analysis
PHILLS	Portable Hyperspectral Imager For Low Light Spectroscopy
PIF	Pseudo-Invariant Feature
QAA	Quasi-Analytical Algorithm
RGB	Red-Green-Blue
RMS	Root Mean Square
SAM	Spectral Angle Mapper
SAMBUCA	Semi-Analytical Model for Bathymetry, Un-mixing and Concentration Assessment
SDI	Substratum Detectability Index
SPOT	Satellite Pour l’Observation de la Terre
SST	Sea Surface Temperature
TM	Thematic Mapper
TOA	Top-of-Atmosphere
UV	Ultraviolet
WV02	WorldView-2 sensor
XS	Multi-spectral

**Appendix A2.** Symbols used in this review.

Symbols	Description	Units
*a*	Total absorption coefficient	m^−1^
*a_CDOM_*	Absorption coefficient of CDOM	
*a_d_*	Absorption coefficient of detritus	
*a_g_*	Absorption coefficient of CDOM and detritus	m^−1^
*a_phyto_*	Absorption coefficient of phytoplankton pigments	m^−1^
*a_w_*	Absorption coefficient of pure water	m^−1^
*b*	Scattering coefficient	m^−1^
*b_b_*	Backscattering coefficient	m^−1^
*b_bp_*	Backscattering coefficients of suspended particles	m^−1^
*b_bw_*	Backscattering coefficients of seawater	m^−1^
*B_ba_*	Substrate weighting coefficients for brown algae	
*B_sg_*	Substrate weighting coefficients for seagrass	
*B_sd_*	Substrate weighting coefficients for sediments	
*c*	Beam attenuation coefficient	m−1
*d*	Distance Sun-Earth at the day of imagery	km
*d*_0_	Mean distance Sun-Earth	km
$D_{u}^{B}$	Length optical path factor from substrate	
$D_{u}^{C}$	Length optical path factor from water column	
*E_d_*	Downwelling irradiance	W·m^−2^·sr^−1^·μm^−1^
*E_do_*	Downwelling irradiance below surface	W·m^−2^·sr^−1^·μm^−1^
*E_dz_*	Sub-aquatic downwelling irradiance at bottom depth	W·m^−2^·sr^−1^·μm^−1^
*E_SUN_*	Solar downward irradiance	W·m^−2^·sr^−1^·μm^−1^
*E_u_*	Upward irradiance	W·m^−2^·sr^−1^·μm^−1^
*f*	Average of the cosine of the scattering angle for phase scattering function	
*g_bm,sand_*	Fractional cover of substrates brown mud and sand within each pixel	
*h_b_*	Constant suitably chosen to tuning the algorithm in the appropriate range	
*K_d_*	Diffuse attenuation coefficient of downward irradiance	m^−1^
*L*	Radiance	W·m^−2^·sr^−1^
*L_b_*	Bottom radiance	W·m^−2^·sr^−1^
*L_w_*	Water-leaving radiance for shallow waters	W·m^−2^·sr^−1^
*L_u_*	Upward radiance	W·m^−2^·sr^−1^
*L_TOA_*	Radiance at the Top-of-Atmosphere	W·m^−2^·sr^−1^
*L_TOA,_*_∞_	Radiance at the Top-of-Atmosphere for deep water	W·m^−2^·sr^−1^
*L*_∞_	Water-leaving radiance for deep water	W·m^−2^·sr^−1^
*m_i_*	Constant that includes the solar irradiance, transmittance of the atmosphere and water surface refraction.	
*n*	Exponent of backscattering by small particles	
*N*	Number of spectral bands	
*q*	Geometric factor that considers length path in water column	m
*Q*	Ratio of the subsurface upward irradiance to radiance conversion factor	
*R*	Irradiance reflectance	
*R_i_*	Irradiance reflectance in band i of shallow water	
*R_ba_*	Brown algae irradiance reflectance	
*R_sd_*	Sediments irradiance reflectance	
*R_sg_*	Seagrass irradiance reflectance	
*R_w_*	Irradiance reflectance of shallow water	
*R*_1_	Photons that do not strike the bottom	
*R*_2_	Photons that strike the bottom once	
*R*_∞_	Irradiance reflectance of deep water	
*s*	Ratio of the number of photons that strike the bottom twice by the number striking once, cited in Gordon and Brown 1974	
*t*	Atmospheric transmittance	
*z*	Depth	m
*z*_90_	Effective penetration depth of imagery	m
[*z* − 0]	Thickness of water layer between depth (*z*) and surface (0)	m
*β_w_*(90°,λ_0_)	Volume scattering function for pure water, at the reference wavelengths 350 and 600 nm (Morel 1974)	m^−1^·sr^−1^
Δ*z*	Intrinsic methodological depth error, Bierwirth *et al.* (1993)	m
ρ*_b_*	Bottom reflectance	
$\rho_{b}^{RS}$	Remote sensing reflectance of bottom	sr^−1^
$\rho_{\infty }^{RS}$	Remote sensing reflectance of deep water	sr^−1^
ρ*_b,i_*	Bottom reflectance in band *i*	
ρ*_bm_*	Reflectance of brown mud	
ρ*_sand_*	Reflectance of sand	
ρ*_w_*	Reflectance of shallow water	
ρ*^RS^*	Remote sensing reflectance	sr^−1^
ρ*_TOA_*	Reflectance at the Top-of-Atmosphere	
ρ_∞_	Reflectance of optically deep water	
ρ*_w_*	Reflectance of shallow water	
θ*_v_*	Water-sensor angle	rad
θ*_s_*	Solar zenith angle	rad
λ	Wavelength	nm
λ_0_	Wavelength selected from the reference wavelength table (Morel 1974)	nm
ψ_1_	Scattering angle between the forward direction of the incident beam and the straight line connecting the detector and the scattering point C_1._ C_1_ corresponds to intersect between water layer surface and beginning of sensor swath, which depends on field of view (FOV)	rad
ψ_2_	Scattering angle between the forward direction of the incident beam and the straight line connecting the detector and the scattering point C_2_. C_2_ corresponds to intersect between water layer surface and end of sensor swath, which depends on field of view (FOV)	rad
0^−^	Just below surface	
0^+^	Just above surface	
